# The genus *Vipio* Latreille (Hymenoptera, Braconidae) in the Neotropical Region

**DOI:** 10.3897/zookeys.925.48457

**Published:** 2020-04-08

**Authors:** Donald L. J. Quicke, Scott R. Shaw, Mian Inayatullah, Buntika A. Butcher

**Affiliations:** 1 Integrative Ecology Laboratory, Department of Biology, Faculty of Science, Chulalongkorn University, Phayathai Road, Pathumwan, BKK 10330, Thailand Chulalongkorn University Pathumwan Thailand; 2 Department of Ecosystem Science and Management, University of Wyoming, Laramie, Wyoming 82071-3354, USA University of Wyoming Laramie United States of America; 3 Department of Entomology, Faculty of Crop Protection Sciences, NWFP Agricultural University, Peshawer, Pakistan NWFP Agricultural University Peshawer Pakistan

**Keywords:** *
Isomecus
*, new species, re-description, South America, systematics

## Abstract

The genus *Vipio* Latreille is revised for the Neotropical region (south of Nicaragua). All species are fully illustrated. Thirteen species are recognised of which five (*V. boliviensis*, *V. carinatus*, *V. godoyi*, *V. hansoni*, and *V. lavignei*) are described as new, all with descriptions attributable to Inayatullah, Shaw & Quicke. All previously described Neotropical species are redescribed. A key is included for the identification of the *Vipio* species known from the Americas south of Nicaragua, and all species are illustrated.

## Introduction

The braconid genus *Vipio* Latreille is most diverse in the Holarctic Region but has a significant representation in the Neotropical Region. However, little is known of these tropical species, only a very few of which are described (Brullé 1846, Ashmead 1900, Szépligeti 1906, Bréthes 1909, 1913), nor are there any host records for the species from this part of the world. Because of a recent upsurge in Hymenoptera studies in South America, the publication of keys to the genera of New World Braconidae (Wharton et al., 1997), and a growing number of biodiversity studies, there is a need to provide identification keys to the Neotropical fauna. Here we present a revision of the nine *Vipio* species now known to occur in South America and southern Central America (south of Nicaragua), which includes descriptions of four new species, and redescriptions of the five previously described ones whose original descriptions do not mention many important characters. An illustrated key to the species is also provided. The Nearctic species were revised (Inayatullah et al., 1997) and since then only one additional New World species, *V. porteri*Inayatullah et al., 2015, has been described. Species from northern Central America, Mexico, and the Caribbean are diverse and comprise a complex assemblage that will be treated in a subsequent paper.

## Materials and methods

### Terminology and collections

Terminology follows van Achterberg (1979, 1988) except for the relative heights of eye (EH) and malar space (MS) follow Inayatullah (1992) and Inayatullah et al. (2012), and wing venation nomenclature which follows Sharkey & Wharton (1997); see also fig. 2.2 in Quicke (2015) for comparison of wing venation naming systems. Sculpture terminology follows Harris (1979). The following abbreviations are used to save space:

**EH** eye height;

**FH** face height measured between anterior margin of antennal socket and anterior tentorial pit;

**FW** face width;

**HH** head height as least distance between base of mandible and lateral ocellus (inclusive), in lateral view;

**HW** head width;

**HL** head length:

**ITD** inter-tentorial distance;

**LMC** labiomaxillary complex;

**LRC** length of radial cell measured between apex of pterostigma and 3RSb;

**MS** malar space;

**PL** pterostigma length;

**PW** maximum width of pterostigma;

**T** tergite;

**TOD** tentorio-ocular distance.

Collections from where specimens were borrowed are abbreviated as follows:

**BMNH**Natural History Museum, London, U.K.;

**CNCI**Canadian National Collection of Insects, Ottawa;

**EMUS**Entomology Museum, Utah State University, Logan (formerly American Entomological Institute, Gainesville);

**ESUW**Entomology Section, University of Wyoming, Laramie, WY;

**HNHM**Hungarian Natural History Museum, Budapest;

**IFML**Tucuman, Instituto Fundación Miguel Lillo, Argentina;

**MACN**Museo Argentino de Ciencias Naturales, Buenos Aires;

**MCZC**Museum of Comparative Zoology, Harvard University, Cambridge, Massachussetts;

**MNHN**Museum national d’Histoire naturelle, Paris;

**USNM**United States National Museum, Washington D.C..

Images were captured with a 3 MP Leica video camera on a Leica M205C stereomicroscope running Leica Application Suite (LAS) software (Leica Microsystems GmbH, Wetzlar, Hesse, Germany), and focus-stacked using the same software. Some minor adjustments in images and plate preparation were performed in Adobe Photoshop version CS6 (Adobe Systems Inc., San Jose, California, United States of America).

## Taxonomy

### Vipio

Taxon classificationAnimaliaHymenopteraBraconidae

Latrielle, 1804

7AAF51E9-1B27-5DF1-9E45-58D16385A3F5

Vipio Latreille, 1804. Nouv. Dict. Hist. Nat. 24: 173. Type-species: Ichneumon desertor Fabricius. Desig. by Foerster, 1862.
Isomecus Kriechbaumer, Prog. Staats-Gym. Pola, 1895: 12. Type-species: Isomecus schlettereri Kriechbaumer, 1895. Synon. by Quicke & Sharkey (1989).
Zavipio Viereck, 1914. U. S. Natl. Mus. Bull. 83: 156. Type-species: Vipio marshalli Schmiedeknecht (Orig. desig.); unnecessary replacement name for Vipio.

#### Remarks.

Members of the genus *Vipio* can be recognised using the keys to genera of Quicke (1987, 1997) or Quicke & Sharkey (1989).

### Key to females of Neotropical species of *Vipio*

**Table:** 

1	Claw with small, rounded basal lobe, at most with very small angulation, but never protruding (a)	**2**
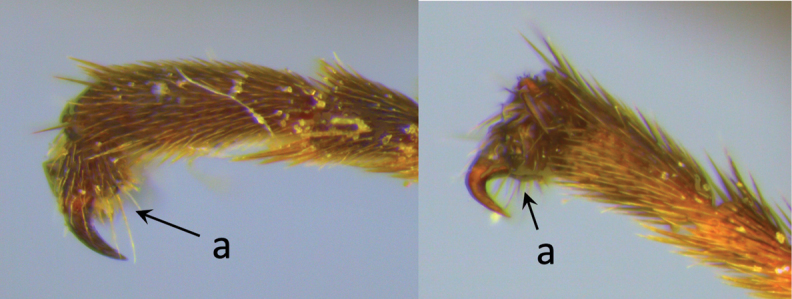		
–	Claw with large, acutely pointed or square basal lobe (b)	**5**
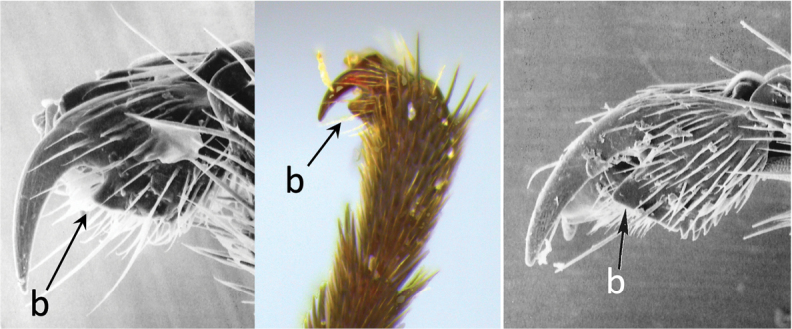		
2	Ovipositor long, exserted part, always more than 0.9 × length of fore wing (a)	**3**
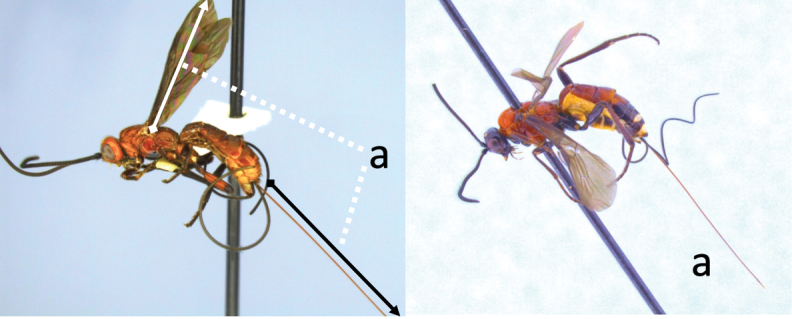		
–	Ovipositor shorter, exserted part less than 0.7 × length of fore wing, usually less than 0.5 × (aa)	**4**
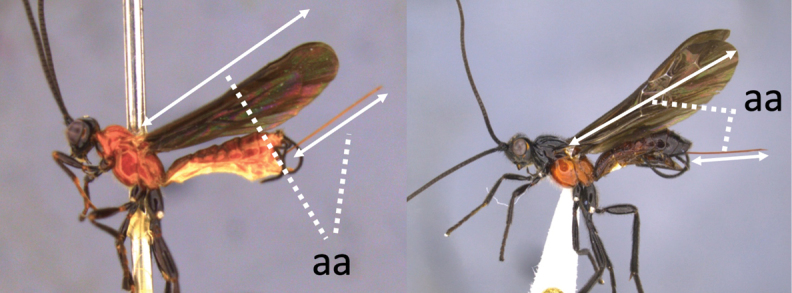		
3	Head largely black (a); base of metasomal T II with 3 smooth triangular areas margined posteriorly by sharp carinate borders (b); hypopygium short, not or hardly extending beyond apex of metasomal tergites (c)	***V. boliviensis* sp. nov.**
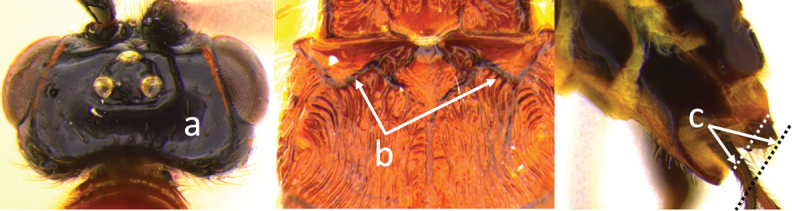		
–	Head orange-red (aa); base of metasomal T II and III without such distinct triangular areas, posterior borders not so evenly or completely carinate, and inside areas of triangles more roughly sculptured, not completely smooth (bb); hypopygium extending beyond apex of metasoma by 0.3–0.7 mm (cc)	***V. belfragei* (Cresson)**
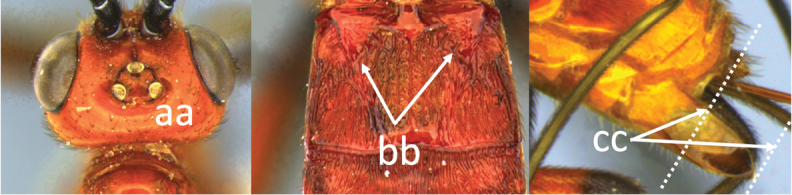		
4	Face largely smooth with weak corrugation (a); second metasomal suture occupying approximately one third length of T III (b); pronotum, mesoscutum and metasomal tergites orange-red (c)	***V. hansoni* sp. nov.**
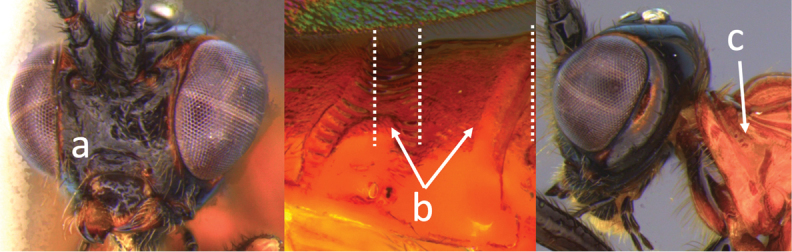		
–	Face strongly sculptured, rugose punctate (aa); 2^nd^ metasomal suture very wide occupying approximately half length of T III (bb); pronotum, mesoscutum and posterior metasomal tergites piceous or black (cc)	***V. lavignei* sp. nov.**
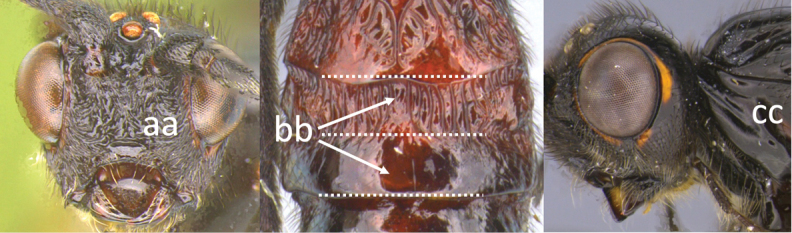		
5	Ovipositor shorter than fore wing (a)	**6**
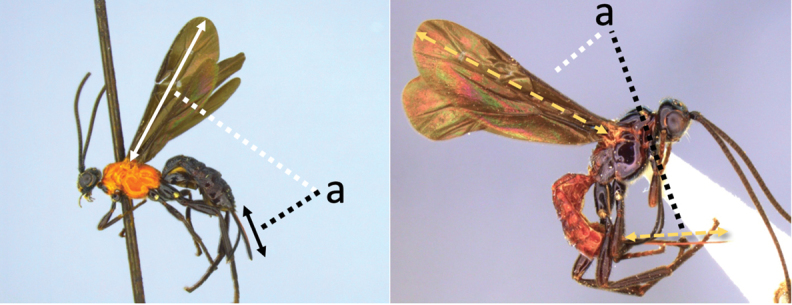		
–	Ovipositor at least 1.3 × fore wing length (aa)	**10**
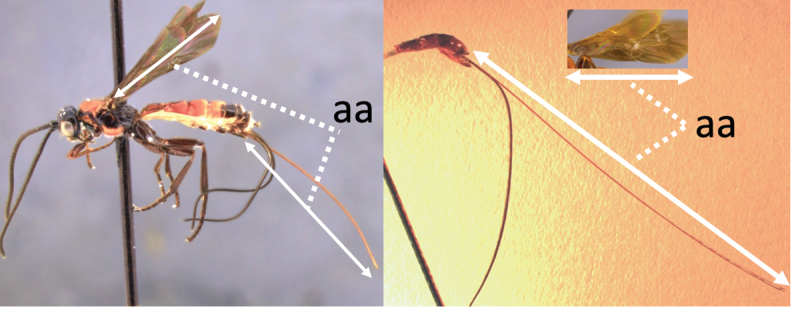		
6	Metasoma entirely black (a)	***V. quadrirugulosus* (Enderlein)**
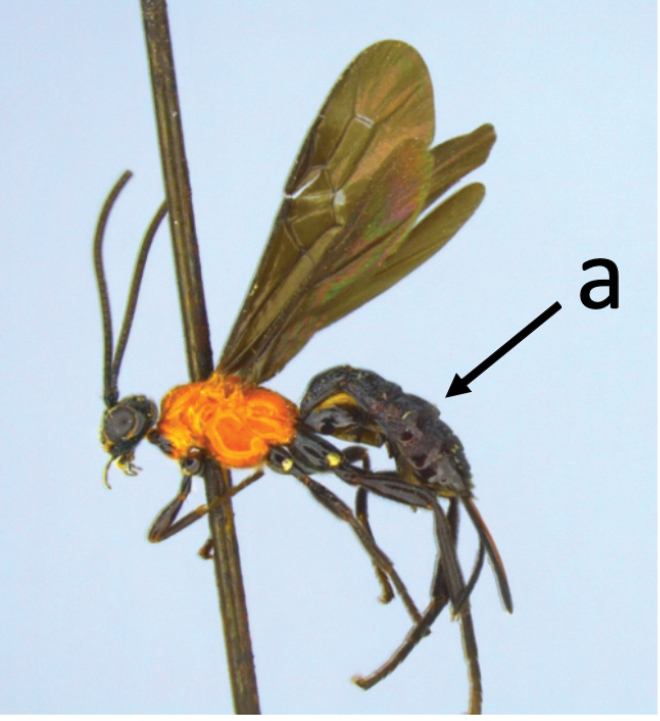		
–	Metasoma largely red (aa)	**7**
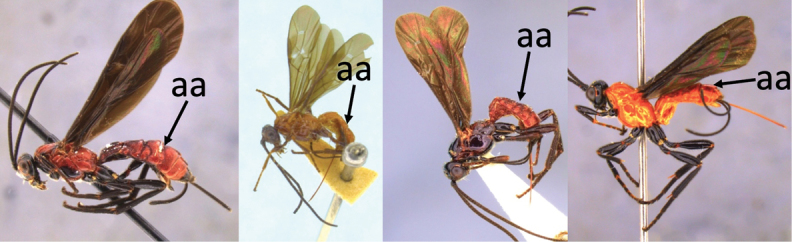		
7	Propodeum with a single, mid-longitudinal anteriorly blunt carina and only a short pair of submedial carinae posteriorly (a)	***V. carinatus* sp. nov.**
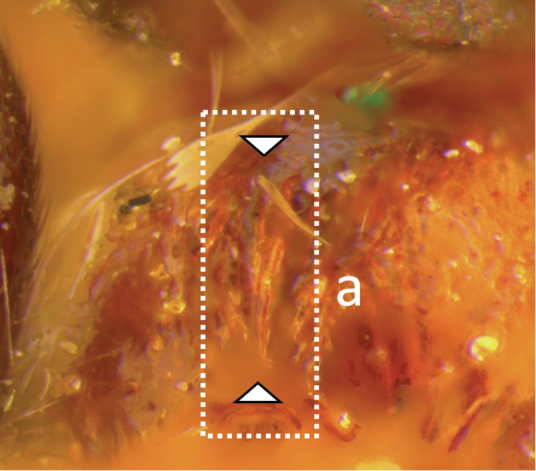		
–	Propodeum smooth (aa) or with numerous anteriorly diverging carinae on posterior half (aaa)	**8**
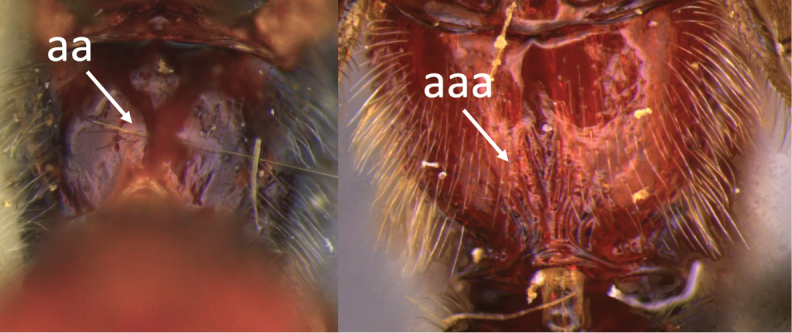		
8	Propodeal spiracle large, > 0.5 (0.56) diameter of median ocellus (a); spiracle of metasomal T III large, > 0.5 (0.57) diameter of median ocellus; dorsolateral carinae of metasomal T I strongly lamelliform (b)	***V. godoyi* sp. nov.**
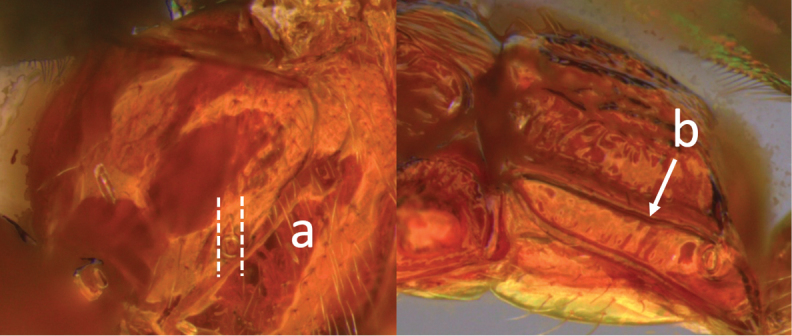		
–	Propodeal and metasomal spiracles smaller (aa); dorsolateral carinae of metasomal T I relatively less developed (bb)	**9**
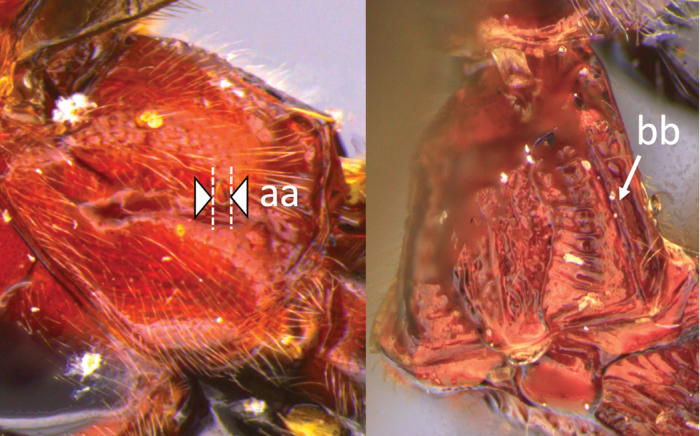		
9	Propodeum with prominent striations posteriorly (a); mesonotum largely reddish (b)	***V. strigator* (Bréthes)**
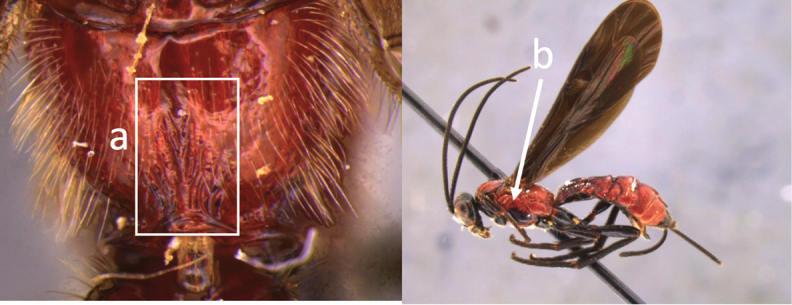		
–	Propodeum smooth (aa); mesoscutum and most of mesosoma reddish black (bb)	***V. thoracica* (Ashmead)**
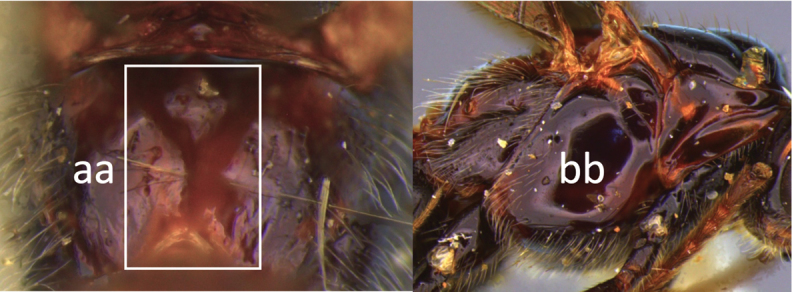		
10	Propodeum with a short median longitudinal wide carina anteriorly (a); ovipositor 1.1–1.4 × body length (b)	***V. paraguayensis* Szépligeti**
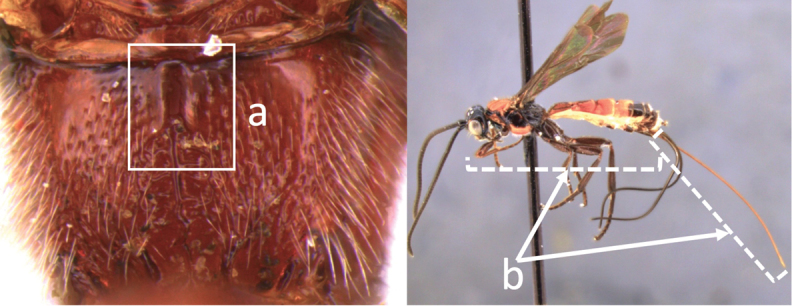		
–	Propodeum without median longitudinal carina anteriorly, with reticulate to areolate rugose sculpture posteriorly (aa, aaa); ovipositor > 1.55 × body length (bb)	**11**
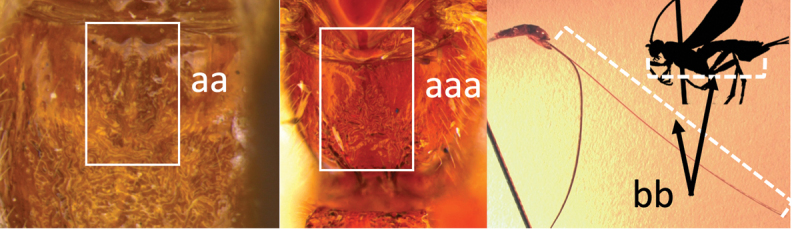		
11	Head yellow or reddish yellow (a)	***V. fiebrigi* Bréthes**
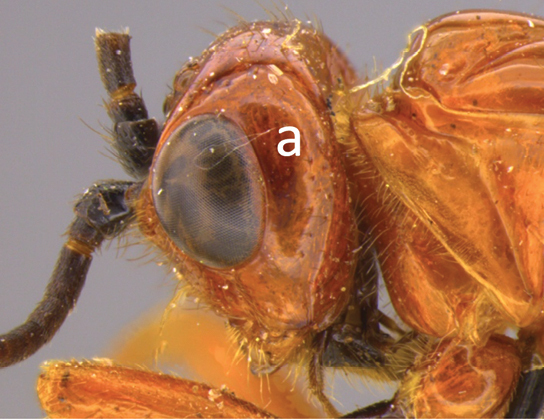		
–	Head black or with extensive black markings dorsally (aa, aaa, aaaa)	**12**
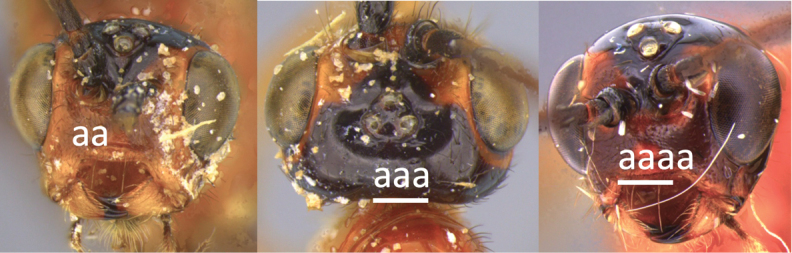		
12	Ovipositor less than 1.9 × body length (range 1.5–1.85); MS less than 0.35 (> 0.31) × maximum eye height in lateral view (a)	***V. porteri* Inayatullah et al.**
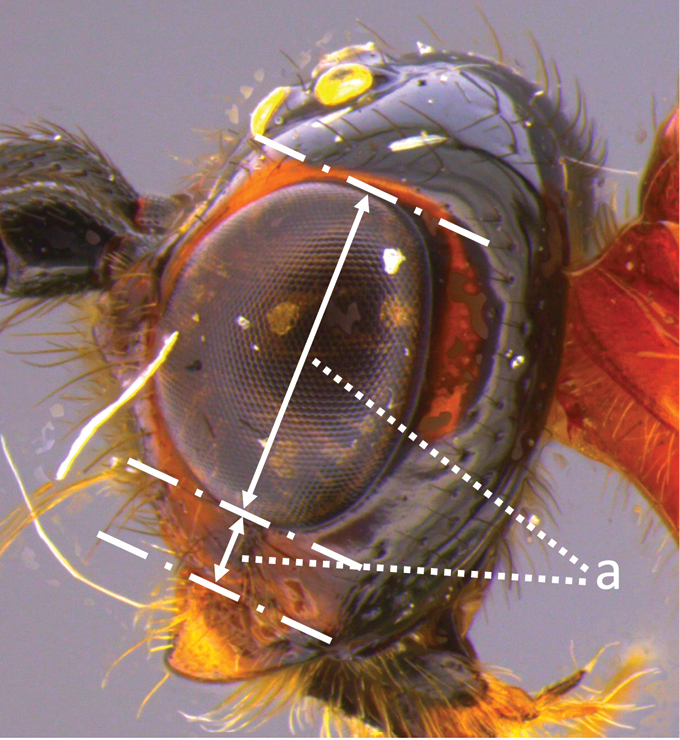		
–	Ovipositor more than 1.9 × body length (range 1.94–2.34); MS more than 0.35 (0.4) × maximum eye height in lateral view (aa)	***V. melanocephalus* Brullé**
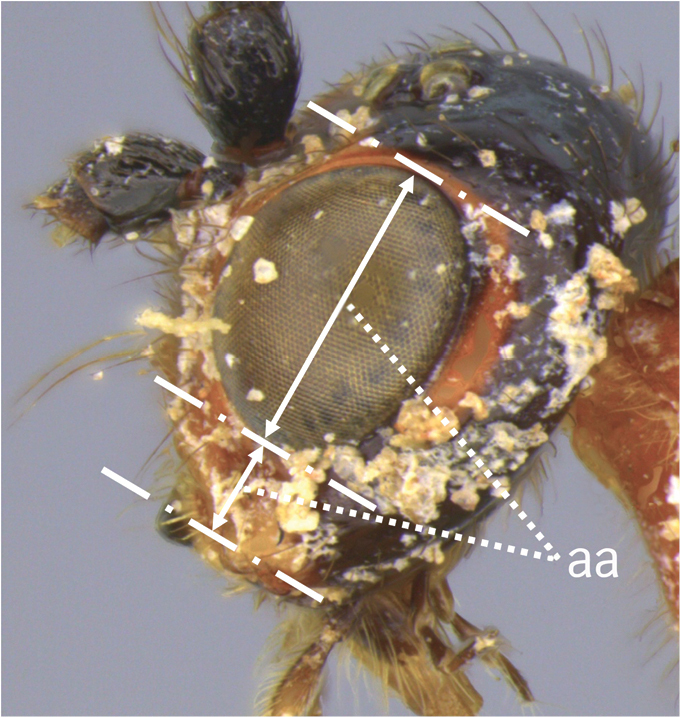		

### Vipio belfragei

Taxon classificationAnimaliaHymenopteraBraconidae

(Cresson, 1872)

4819422C-3FF3-5CA1-A9E0-196405F5C0F5

Figures 1 , 2

Bracon belfragei Cresson, 1872: 186; Vipio belfragei: Pierce, 1908: 44; Shelefelt, 1978: 1843; Inayatullah et al., 1998: 125–127, figs 3, 25; López-Martínez et al. 2009: 215; Zavipio belfragei: Sattertwait, 1932: 1003.

#### Type material.

Holotype ♀, **USA**, Texas, (no date), W. Belfrage (USNM type No. 1610) (examined).

#### Comments.

Additional material examined is summarised in Inayatullah et al. (1998) who re-described it. It is fully illustrated here for the first time. This is a common, widespread, and rather variable (Inayatullah et al. 1998) species in the USA and Mexico with a range extending as far south as Costa Rica and Panama.

**Figure 1.**
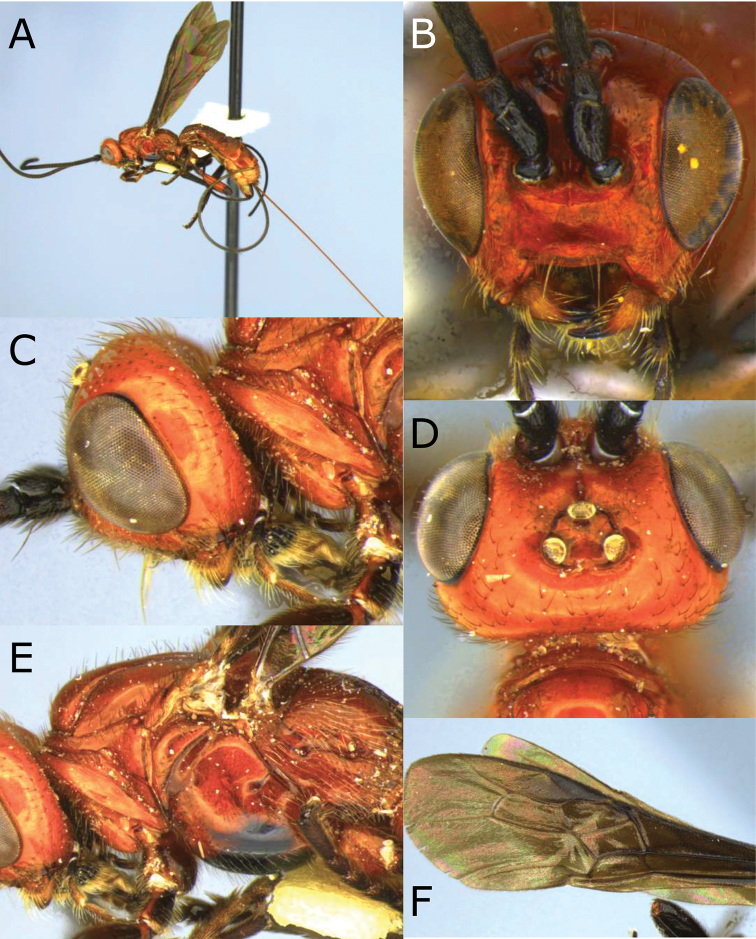
Montaged light micrographs of *Vipio belfragei* female. **A** Habitus, lateral view **B** face **C** head and anterior mesosoma, lateral view **D** head, dorsal view **E** mesosoma, lateral view **F** wings **G** claw.

**Figure 2.**
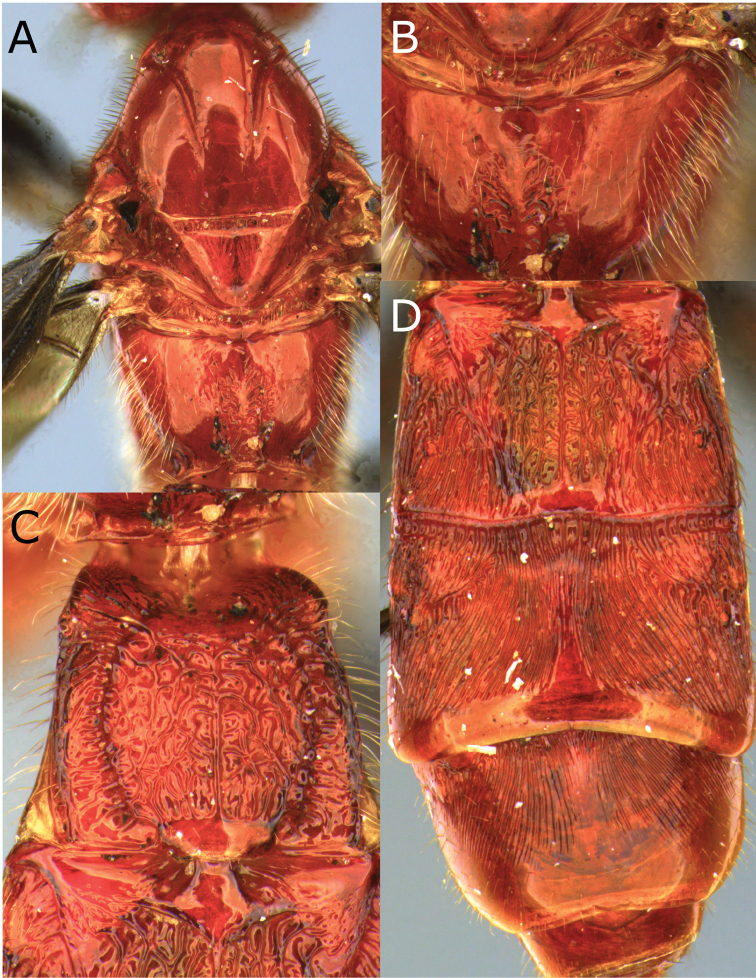
Montaged light micrographs of *Vipio belfragei* female. **A** Mesosoma, dorsal view **B** propodeum **C** metasomal tergite I, near dorsal view **D** metasomal tergites II–IV, dorsal view.

### Vipio boliviensis sp. nov.

Taxon classificationAnimaliaHymenopteraBraconidae

1ACD4C45-9173-5B1A-8167-E560BC1E4C8D

http://zoobank.org/30868A9A-2247-4249-B01A-78FC9612222D

Figures 3 , 4

#### Type material.

Holotype ♀, **Bolivia**: Comarapa, 18 m., 14.xii.1984 (L. Pena) (EMUS). Paratype: **Argentina**: 1 ♀, Pronunciamiento Entre Rios, xii.1965 (CNCI); 1 ♀, E. Rios, xii.1972, Feliciano Fritz col. (EMUS).

#### Diagnosis.

Can be distinguished from other Neotropical *Vipio* species by the combination of a hypopygium ending at apex of metasoma and a long ovipositor (ovipositor length/body length 1.17). Additionally, it has claws without basal lobe, ovipositor longer than fore wing.

#### Description.

**Female.** Length of body 5.4 mm; of fore wing 5.6 mm; of ovipositor (part exserted beyond apex of abdomen) 6.4 mm.

***Head.*** Antenna robust, with 37–41 flagellomeres; terminal flagellomere blunt and distinctly laterally compressed; median flagellomeres as long as wide, more distal flagellomeres becoming thicker; first flagellomere 1.5 × longer than second, 3.3 × longer than wide; second flagellomere 2.2 × longer than wide; head transverse; HL 0.78 × HH; clypeus rugulose; clypeal guard setae typical; face slightly punctate; remainder of head smooth and shiny; HW/HH 0.96; FH/FW 0.5; EH/HH 0.73; EH/FW 1.0; ITD 2.25 × TOD; MS 0.25 × EH; LMC slightly less than 0.5 × HH; third segment of maxillary palpus 5 × longer than wide.

***Mesosoma.*** Length of mesosoma 1.43 × height. Pronotum carinate antero-laterally. Notauli smooth. Propodeum rugulose medially, with a blunt and short median longitudinal carina not reaching posterior end, and with a pair of short, longitudinal, anteriorly diverging submedial carinae not reaching middle of propodeum; remainder of mesosoma laterally smooth and shiny.

***Wings.*** Fore wing: length of fore wing 1.03 × body length; PL/LRC 0.75; PW/PL 0.19; length of vein 1M 0.71 × length of (RS+M)a; length of vein 3RSb 0.82 × combined length of r-rs and 3RSa; vein 3RSa reaching wing margin 0.55 × distance between apex of pterostigma and wing tip. Hind wing: with a glabrous area distal to cu-a; apex of C+SC+R with one basal hamule

***Legs.*** Claw without pointed basal lobe.

***Metasoma.*** First metasomal tergite 1.20 × longer than wide, raised median area of T I oval, rugose, with a median longitudinal carina posteriorly, surrounding area with short, transverse carinae, dorso-lateral carina present; T II 1.5 × wider than long medially, longitudinally striate, basal areas rugulose, oblique furrow strongly impressed; T III 1.45 × wider than long medially, anteriorly with longitudinal striations running postero-laterally, and with transverse striations posteriorly; T IV entirely with transverse striation but not reaching lateral margin which is smooth and shiny; T V–VII smooth and shiny; hypopygium short, ending at apex of metasoma; ovipositor sheath with sparse setae; ovipositor 1.17 × body length.

***Colour.*** Yellowish red except head, antenna, palpi, prosternum, T IV posteriorly, and T V–VII, metasomal laterotergites and ovipositor sheath black. Wings smoky.

**Male.** Unknown.

#### Remarks.

This species appears to be closely related to *V. paraguayensis* Szépligeti, because of the presence of a median longitudinal carina on propodeum and similar sculpture of T1 and T2. *Vipio boliviensis* can be distinguished by the carinate pronotum (smooth and shiny in *paraguayensis*), absence of pointed basal lobe on the claw (present in *paraguayensis*), transverse striations on T III and T IV (longitudinal in *paraguayensis*), and short hypopygium (long in *paraguayensis*).

#### Etymology.

Named after the country of Bolivia, where the holotype was collected. We have retained this name despite the recent discovery of a specimen from Argentina because of its use in Inayatullah’s MS thesis (1992).

**Figure 3.**
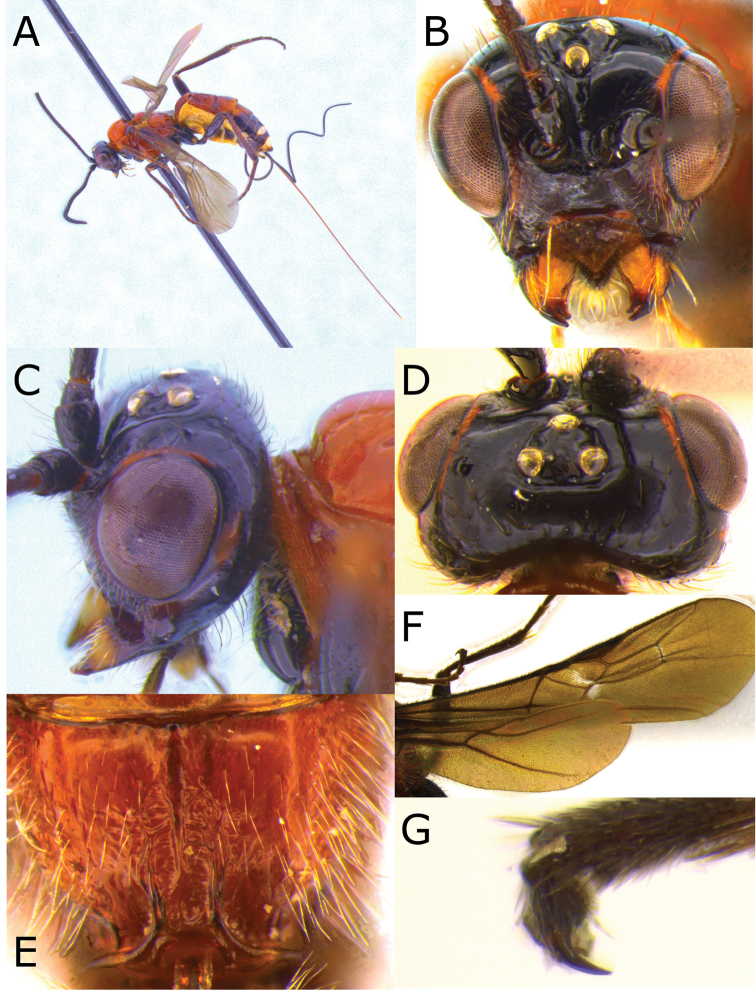
Montaged light micrographs of *Vipio boliviensis* sp. nov. **A** Holotype, habitus lateral view **B** head, front view **C** head, oblique view **D** head, dorsal view **E** propodeum **F** wings **G** claw.

**Figure 4.**
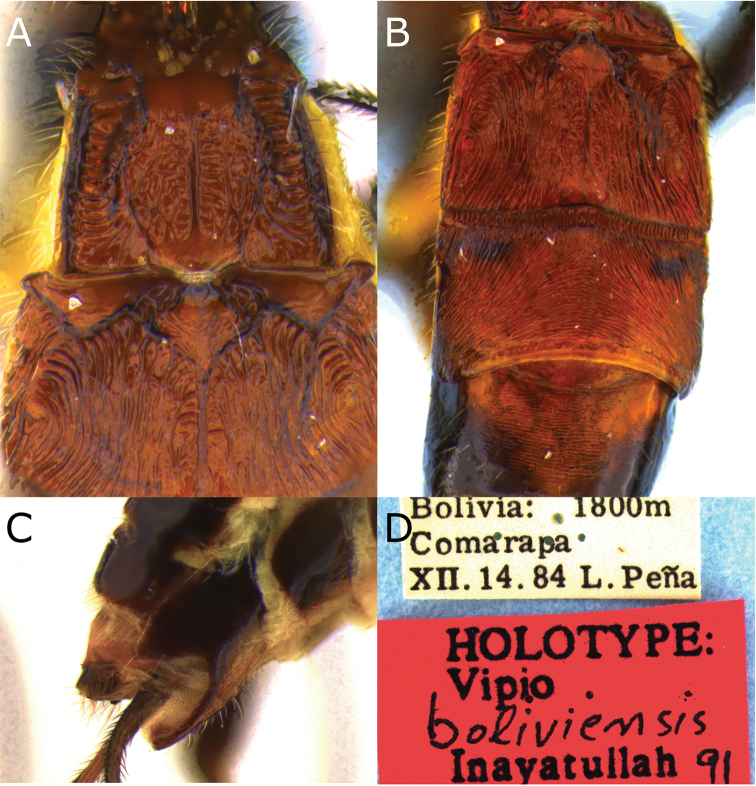
Montaged light micrographs of *Vipio boliviensis* sp. nov. **A** Metasomal tergites I and II **B** metasomal tergites II–IV **C** apex of metasoma, lateroventral view **D** holotype specimen labels.

### Vipio carinatus sp. nov.

Taxon classificationAnimaliaHymenopteraBraconidae

57552F98-4048-5BAD-8519-3CF42FD64245

http://zoobank.org/B63E1D1A-8159-4C1D-9DBC-D8B0EE61D5B4

Figures 5 , 6

#### Type material.

Holotype ♀, **Bolivia**: Sara (no date), (Steinbach) (MCZC). Paratypes: **Bolivia**: Santa Cruz, 1 ♀, 28.i–ii.1964 (Z. Golbach) (IFML); 1 ♂, (no date) (J. Steinbach) (MCZC). **Argentina**: 1 ♂, Chaco, Colonia, Benítez, 10.xii.1948 (R. Golbach) (IFML).

#### Diagnosis.

Ovipositor shorter than fore wing, propodeum with an anteriorly blunt but complete mid-longitudinal carina, claw with pointed basal lobe, ovipositor approximately half length of fore wing, body except head, predominantly yellow.

#### Description.

Holotype ♀ length of body 4.6–5.0 mm, of fore wing 4.6–5.0 mm and of ovipositor (part exserted beyond apex of abdomen) 2.5 mm.

**Figure 5.**
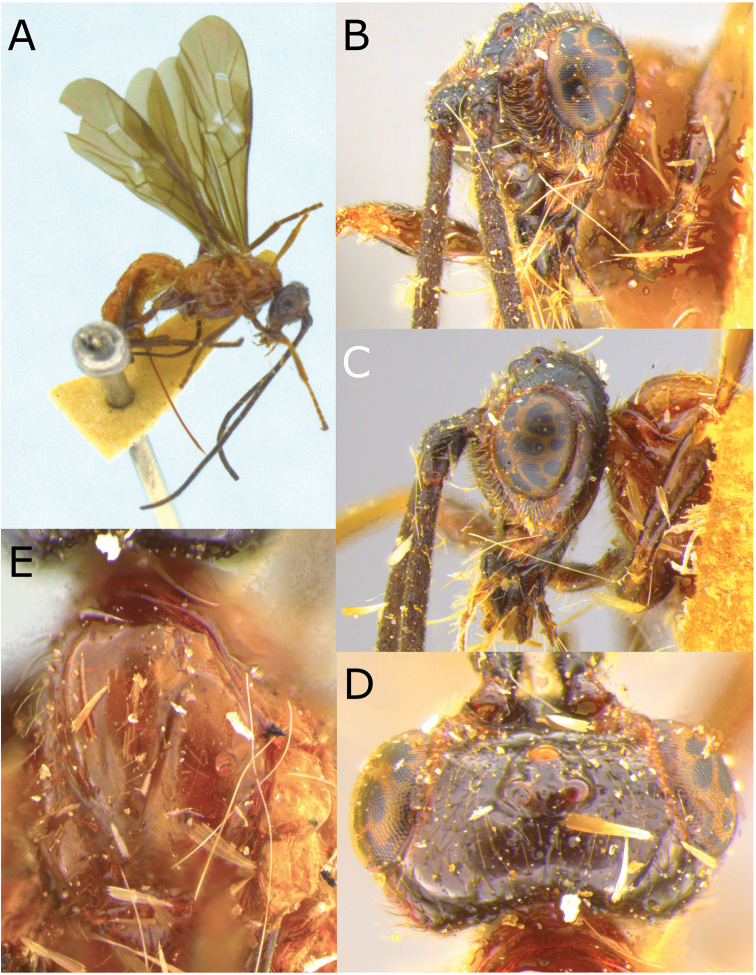
Montaged light micrographs of *Vipio carinatis*. **A** Holotype, habitus lateral view **B** face, oblique view **C** head, lateral view **D** head, dorsal view **E** mesoscutum, oblique dorsal view.

***Head.*** Antenna robust, 1.1 × body length, with 42 flagellomeres; first flagellomere 1.4 × longer than second, 1.1 × longer than wide; second flagellomere 1.2 × longer than wide; flagellomeres beyond the fifth 1.1–1.2 × longer than wide; median flagellomeres slightly shorter than wide; terminal flagellomere sharply pointed apically; head transverse; HL 0.79 × HH; clypeal guard setae consist of one long and one short seta near each anterior tentorial pit; clypeus rugulose; face rugulose, with a median and slightly raised triangular area above the clypeus; remainder of head smooth and shiny; HW/HH 0.77; FH/FW 0.55; EH/HH 0.65; EH/FW 0.92; EW/EH = 0.75; ITD 1.85 × TOD; MS 0.37–0.42 × EH (Fig. 5B); LMC 0.5 × HH; third segment of maxillary palpus 6 × longer than wide.

***Mesosoma.*** Length of mesosoma 1.71 × height; pronotal furrow crenulate dorsally and dorso-laterally; notauli smooth; propodeum slightly rugulose with an anteriorly blunt median longitudinal carina and one short longitudinal carina lateral to the median longitudinal carina posteriorly.

***Wings.*** Fore wing : length of fore wing 1.0 × body length; PL/LRC 0.89; PW/PL 0.28; length of vein 1M 0.67 × length of (RS+M)a; length of vein 3RSb 1.0 × combined length of r-rs and 3RSa; vein 3RSa reaching anterior wing margin 0.61 × distance between apex of pterostigma and wing tip. Hind wing: uniformly setose; apex of C+SC+R with 1 basal hamule.

***Legs.*** Claw with pointed basal lobe.

**Figure 6.**
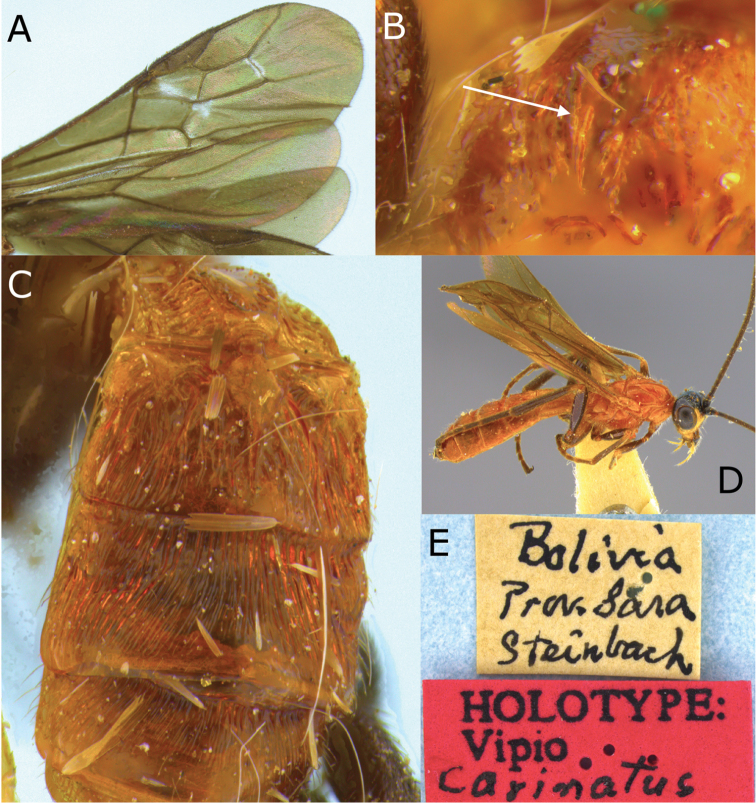
Montaged light micrographs of *Vipio carinatus*. **A** Wings **B** propodeum **C** metasomal tergites I–III, oblique dorsal view **D** paratype male, oblique dorsal habitus **E** holotype specimen labels.

***Metasoma.*** First metasomal tergite 1.2 × longer than wide, raised median area oval, slightly rugulose, with a blunt and irregular dorso-lateral carinae which are more pronounced anteriorly, surrounding area with short transverse carinae, dorso-lateral carina present; T II-IV longitudinally striate (Fig. 6C); T II 1.8 × wider than medially long, basal areas smooth and shiny, oblique furrow impressed; T III 1.7 × wider than medially long; T V–VII smooth and shiny; hypopygium ending at same level as tergites; ovipositor 0.47–0.5 × body length.

***Colour.*** Predominantly yellow to orange-yellow, head and antenna black, except maxillary palp, face laterally, and basal half of mandible blackish red, legs blackish red except fore tibia and tarsi yellow (Fig. 5A). Wings brown with dark brown venation, pterostigma entirely dark brown (Fig. 6A).

#### Variation.

Female paratype as in holotype, except EH/HH 0.68; FH/FW 0.61; EH/FW 1.0; ITD 1.6 × TOD; mesosoma red. Male paratypes (Fig. 6D) as in female, except length of body 6.2–6.5 mm; length of fore wing/body length 0.74–0.82; HL 0.8–0.82 × HH; EH/HH 0.69–0.71; EW/EH 0.70–0.73; EH/FW 1.0–1.04; ITD 1.6–1.9 × TOD; MS 0.28–0.30 × EH. Face yellowish white with a black spot above clypeus; carinae on propodeum more pronounced than in female.

#### Etymology.

Named for the presence of distinctive carinae on the propodeum which are diagnostic.

#### Comments.

Based on the presence of a raised area on face, strongly striate metasoma, and short hypopygium, this species is most closely related to *V. rugator* (Say). The presence of carinae on the propodeum and the long ovipositor (ovipositor length/body length 0.47–0.5) distinguish *V. carinatus* from *V. rugator*, which lacks the carinae on the propodeum and has a shorter ovipositor (ovipositor length/body length 0.29–0.37).

### Vipio fiebrigi

Taxon classificationAnimaliaHymenopteraBraconidae

Bréthes, 1909

5ACB48EB-986B-5688-8476-F0B60BCD6B69

Figures 7 , 8

Vipio fiebrigi Bréthes, 1909: 231; Shenefelt, 1978: 1849; Quicke & Genise, 1994: 44.

#### Type material.

Holotype ♀, *Vipio fiebrigi* Bréthes, 1909, **Paraguay**: San Bernardino (no date) (Fiebrig) (MACN).

#### Additional specimens examined.

**Argentina**: 1 ♀, Chaco, Las Brecias (no date, collector) (USNM); 1 ♀, Chaco, Montevidio So. Amer. Paras lab, No. 674.20, v.1942 (Berry) (USNM); 1 ♂, Chaco, Colonia Benintez, 10.xii.1948 (R. Golbach) (IFML); 3 ♂, Tucuman, Aráoz, Estacion, 8.i.1927 (no collector) (IFML).

#### Diagnosis.

This species can be distinguished from other Neotropical species with very long ovipositors (> 2.0 × body length) by having a yellow-red head, densely striate metasoma and a pointed basal lobe to the claw.

#### Description.

Holotype♀, length of body 8.5–12.3 mm, of fore wing 6.5–9.0 mm, and of ovipositor (part exserted beyond apex of abdomen) 21.5–30.0 mm.

***Head.*** Antenna robust, 0.94–0.97 × body length, with 62–68 flagellomeres; remaining 0.92–1.0 × longer than wide; first flagellomere 1.4 × longer than second; first flagellomere 2.7 × longer than wide; second flagellomere 1.4 × longer than wide; median flagellomeres 1 × longer than wide; terminal flagellomere (missing); head transverse to sub-transverse; clypeus higher in profile, slightly rugulose, clypeal guard setae typical; face sparsely punctate or rugulose; remainder of head smooth and shiny; HL 0.79–0.84 × HH; HW/HH 0.79–0.84; FH/FW 0.42–0.45; EH/HH 0.62–0.64; EH/FW 0.75–0.78; EW/EH 0.7–0.75; ITD 1.5–1.65 × TOD; MS 0.42–0.46 × EH; LMC 0.4 × HH; third segment of maxillary palp 4 × wider than long.

***Mesosoma.*** Length of mesosoma 1.70–1.81 × height; pronotum smooth and shiny or transversely carinate dorso-laterally, smooth and shiny or crenulate at furrow dorso-laterally; notauli smooth, mesonotal lobes well defined; metapleuron smooth to slightly punctate; propodeum strongly reticulate or areolate-rugose postero-medially, smooth or punctate on basal and lateral margins.

**Figure 7.**
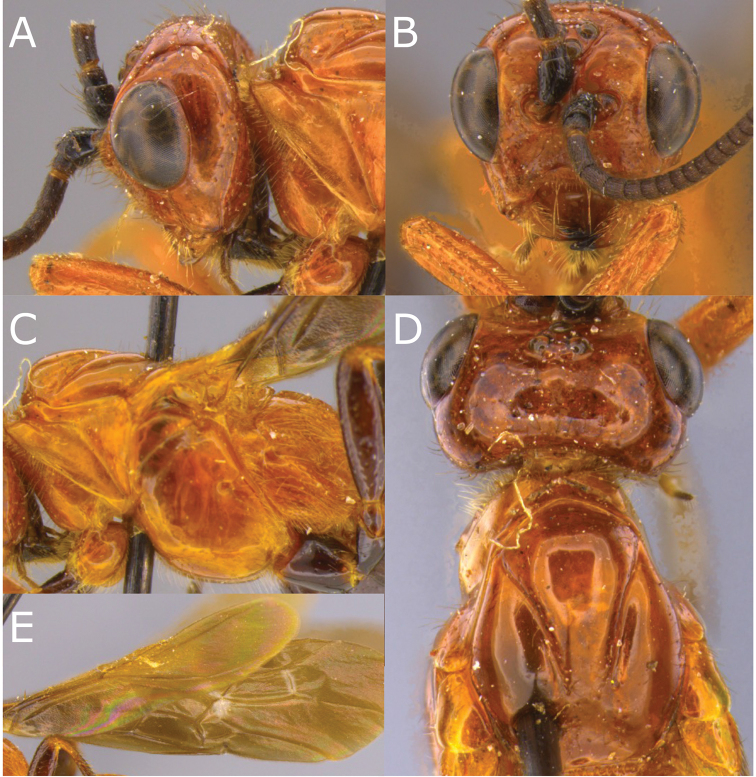
Montaged light micrographs of *Vipio fiebrigi* sp. nov. **A** Head, posterolateral view **B** face **C** mesosoma lateral view **D** head and mesoscutum, dorsal view **E** wings.

***Wings.*** Fore wing: length of fore wing/body length 0.72–0.76; PL/LRC 0.89–0.94; PW/PL 0.21–0.28; length of vein 3RSb 0.82–0.87 × combined length of r-rs and 3RSa; length of vein 1M 0.78–0.80 × length of (RS+M)a; vein 3RSa reaching wing margin 0.52–0.59 × distance between apex of pterostigma and wing tip. Hind wing: with basal glabrous area and/or with sparse basal setosity (Fig. 7E); apex of vein C+SC+R with one basal hamule.

***Legs.*** Claw with strong pointed basal lobe.

***Metasoma.*** First metasomal tergite 1.32–1.34 × longer than wide, rectangular, slightly narrowing anteriorly; raised median area oval, areolate-rugose; basal smooth area narrowing and continuing posteriorly as median longitudinal carina reaching small smooth raised area at the apex of tergum; surrounding area with short transverse carinae; dorso-lateral carina present, area below crenulate; T II 1.15–1.25 × wider than medially long, depressed, baso-lateral areas sub-triangular, smooth and shiny, mediobasal area smooth and shiny, continuing posteriorly as median longitudinal carina, remainder of tergum longitudinally striate, oblique furrow strongly impressed, striate; T III 1.4 × wider than medially long, longitudinally striate, baso-lateral area well defined; T IV longitudinally striate, baso-lateral area short and transverse; T V–VII smooth and shiny; hypopygium extending 0.4–1.0 mm beyond apex of metasoma, ovipositor 2.2–2.6 × body length.

***Colour.*** Yellow to reddish yellow, except tip of mandible, labial palp, basal two segments of maxillary palp, labio-maxillary complex, antenna basally, fore trochanter, middle and hind legs and ovipositor sheath black. slightly smoky to dark brown, pterostigma black, yellow basally.

**Male**. Unknown.

#### Remarks.

Based on the long body, ovipositor length, similar propodeal and metasomal sculpture, this species is closely related to *V. melanocephalus* Brullé. The longer MS (0.42–0.46 × EH) and yellow head in *fiebrigi* will separate it from *melanocephalus*, in which the MS/EH ratio is 0.39–0.41 and the head is black.

**Figure 8.**
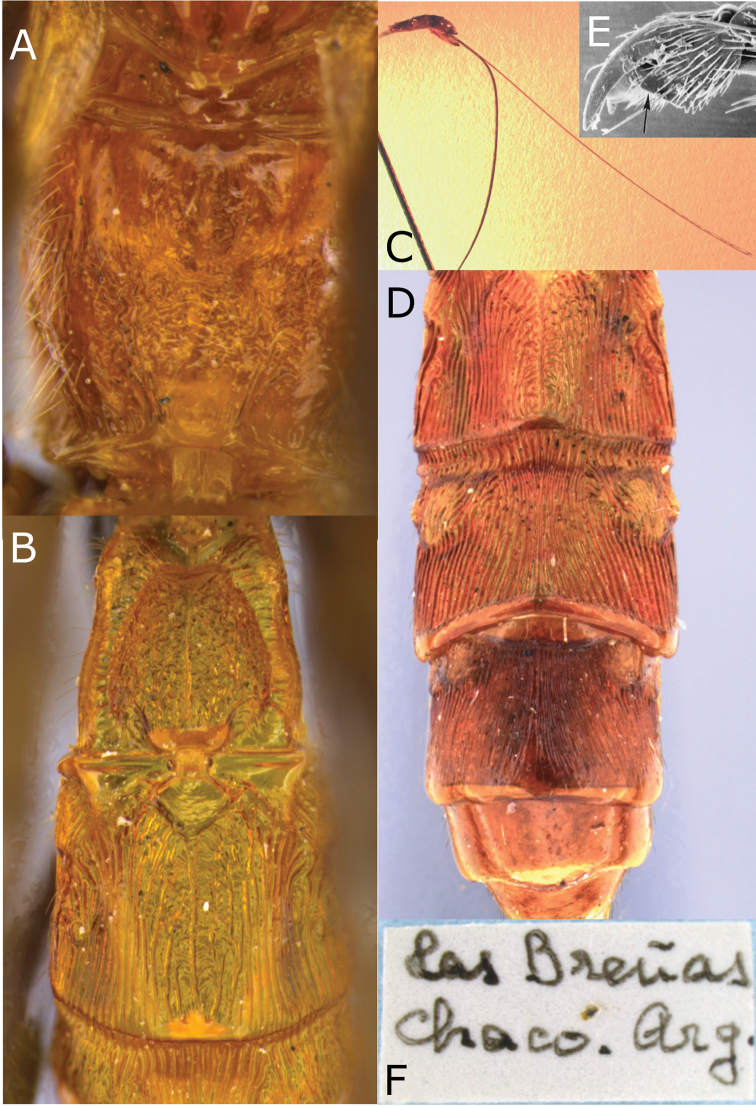
Montaged light and scanning electron micrographs of *Vipio fiebrigi* sp. nov. **A** Propodeum **B** metasomal tergites I and II **C** metasoma, lateral view, showing relative length of ovipositor **D** metasomal tergites II–VI **E** SEM of claw **F** data label of holotype.

### Vipio godoyi sp. nov.

Taxon classificationAnimaliaHymenopteraBraconidae

FE9FC9AF-0566-52DF-8B58-30DAE2D00C48

http://zoobank.org/80C332E5-D2BC-455A-972C-433FDF88E0E8

Figures 9 , 10

#### Type material.

Holotype ♀, **Costa Rica**, Heredia, Chilamate, 75 m, 25.i.-1989 (Hanson & Godoy) (ESUW). Paratypes: **Costa Rica**: 1 ♂, same data as holotype, except 25.iii.1989. 1 ♂, Alajuela, Rio-Laguna Arenal, 500 m, 14.viii.1988 (Paul Hanson). 2 ♂♂, Limon, Rio Toro Amarillo nr. Guapiles, 19.viii.1964 (G.C. Eickwort); 1 ♂, (same data) (USNM); 1 ♂, Heredia, La Selva Res. Sta., 11–17.vi.1986 (W. Hanson, G. Bohart) (EMUS); 1 ♀, same locality, ii-iv.1993 (P. Hanson), huertos Malaise trap set by G. Wright (ESUW). **Honduras**: 1 ♂, Suyapa MorÀzan, 3.xi.1965 (N.L.H. Krauss) (USNM). **Nicaragua**: 1 ♂, Zelaya, El Recreo, x.1984 (no collector) (MCZC). **Panama**: 1 ♂, C.Z. (Canal Zone) Summit, ix.1946 (N.L.H. Krauss) (ESUW); 1 ♂, same data, except (USNM).

#### Diagnosis.

*Vipio godoyi* can be recognised by the combination of large propodeal (Fig. 10A) and metasomal spiracles (Fig. 10C), claw with large pointed basal lobe, strongly laminate T1 dorso-lateral carinae, and short ovipositor and hypopygium.

#### Description.

Holotype ♀ length of body 7.1 mm; fore wing 7.1 mm and of ovipositor 3.8 mm.

***Head.*** Antenna, broken, with 47 flagellomeres remaining, median flagellomeres longer than wide; first flagellomere 2.5 × longer than wide, 1.3 × longer than second, the latter 2.0 × longer than wide; clypeus rugulose, clypeal guard setae typical; face minutely punctate, smooth and shiny; head 0.87 × longer than high; HW/HH 0.8; FH/FW 0.59; EH/HH 0.71; EH/FW 1.04; EW/EH 0.77; ITD 1.8 × TOD; MS 0.3 × EH; third segment of maxillary palpus 3.3 × longer than wide; LMC 0.4 × HH.

***Mesosoma.*** Length of mesosoma 1.7 × height; smooth and shiny; notauli smooth; propodeum smooth, spiracle large, 0.56 × diameter of median ocellus.

***Wings.*** Length of fore wing: 1.0 × body length; PL/LRC 0.8; PW/PL 0.25; length of vein 3RSb 0.88 × combined length of r-rs and 3RSa; length of vein 1M 0.7 × length of (RS+M)a; vein 3RSa reaching anterior wing margin 0.71 × distance between apex of pterostigma and wing tip. Hind wing: uniformly setose; apex of vein C+SC+R with two basal hamules.

***Legs.*** Claw with pointed basal lobe.

***Metasoma.*** First tergite 1.1 × longer than posteriorly wide; raised median area oval, rugulose, with a median longitudinal ridge posteriorly, surrounding area smooth and shiny; dorso-lateral carina laminate, area below smooth and shiny, carina absent above spiracle; T II 1.75 × wider than medially long, longitudinally striate, basal areas smooth and shiny, oblique furrows impressed, striate; T III 1.9 × wider than medially long, longitudinally striate except apex smooth, anterolateral area smooth; all metasomal spiracles large, those of T III 0.57 × the diameter of median ocellus; T IV with short longitudinal striae at base and posterior to anterolateral area, remainder of tergum, smooth and shiny; T V–VII smooth and shiny, mostly retracted; hypopygium barely extending beyond apex of metasoma (Fig. 10B); ovipositor 0.54 × body length.

***Colour.*** Reddish yellow, except head, including mouthparts and antenna, legs and ovipositor sheath black. Wings black.

#### Variation.

Paratype males (*N* = 10) as in female, except body length 7.5–7.9 mm; FWL/BL 0.76–0.83; AL/BL 0.8–0.95; HL/HH 0.8–0.85; EH/HH 0.59–0.62; FH/FW 0.76; EH/FW 0.0.87–0.90; EW/EH 0.75–0.77; ITD 1.64–1.79 × TOD; MS 0.38 × EH; first five flagellomeres 1.8–3.4 × longer than wide; remaining flagellomeres 1.2–1.4 × longer than wide; terminal flagellomere acutely pointed; face smooth and shiny, yellowish white with a black spot above clypeus; third segment of maxillary palpus swollen, 1.9–2.1 × longer than wide; T II-V densely longitudinally striate, striations sometimes absent on posterior part of T V; spiracle of T III of males 0.6–1.0 × the diameter of median ocellus; T VI minutely punctate. Paratype female (*N* = 1) with terminal flagellomere acutely pointed.

#### Biology.

Unknown.

#### Distribution and seasonality.

Costa Rica, Honduras, Nicaragua and Panama. Recorded flying from February through August in Costa Rica, November in Honduras and Panama, and October in Nicaragua. May occur sympatrically with *V. hansoni* sp. nov. (in one case, specimens of both species were taken from the same Malaise trap sample).

#### Comments.

*Vipio godoyi* is apparently closely related to *V. hansoni* sp. nov. based on similar body colour, stout antennae, smooth and shiny propodeum, lamelliform dorsolateral carinae of T I, deeply impressed oblique furrows, oval and posteriorly narrowed raised median area of T I, short hypopygium, and short ovipositors in both species. Females of *V. godoyi* sp. nov. can be separated from those of *V. hansoni* sp. nov. by the presence of a pointed basal lobe on claw (absent in *hansoni*), and the setosity of ovipositor sheath (Fig. 10B). Males of *V. godoyi* have visibly larger and broader spiracles on metasomal T I-III as compared with those of other species. The diameter of spiracle on T III in males of *V. godoyi* is 0.6–1.0 × the diameter of median ocellus (0.35 × in *hansoni*).

#### Etymology.

*Vipio godoyi* is named after Ms. Carolina Godoy, currently of the Instituto Nacional de Biodiversidad (INBio), who assisted with collection of the holotype specimen.

**Figure 9.**
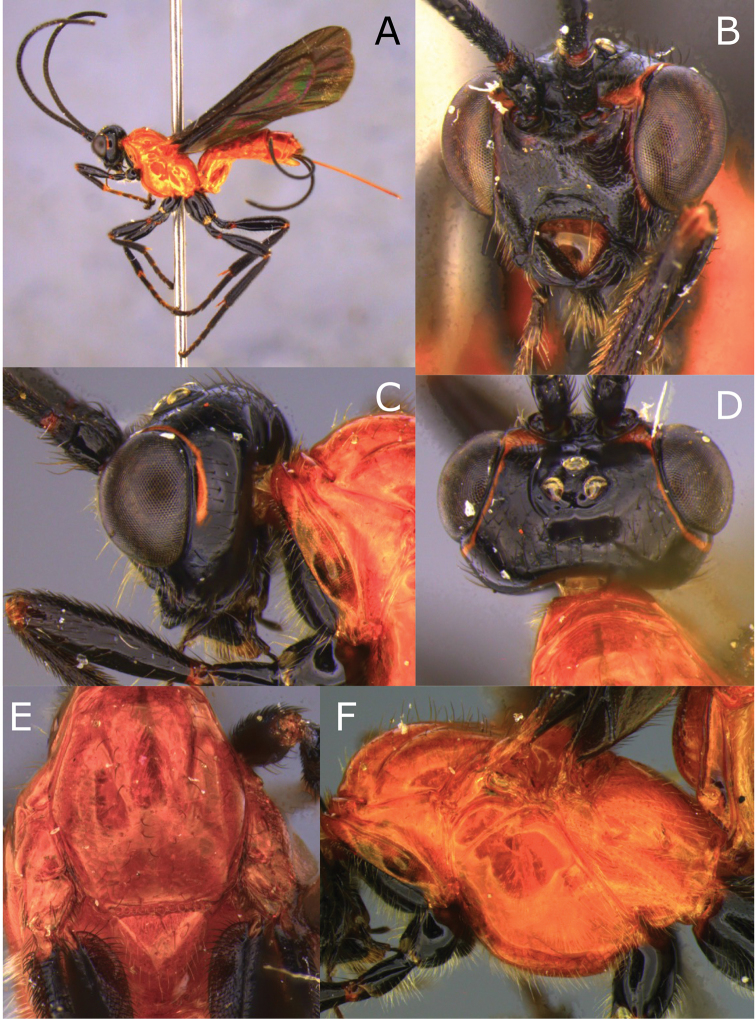
Montaged light micrographs of *Vipio godoyi* sp. nov. **A** Habitus lateral view **B** face **C** head and anterior mesosoma, postero-lateral view **D** head, dorsal view **E** anterior mesosoma, dorsal view **F** mesosoma, lateral view.

**Figure 10.**
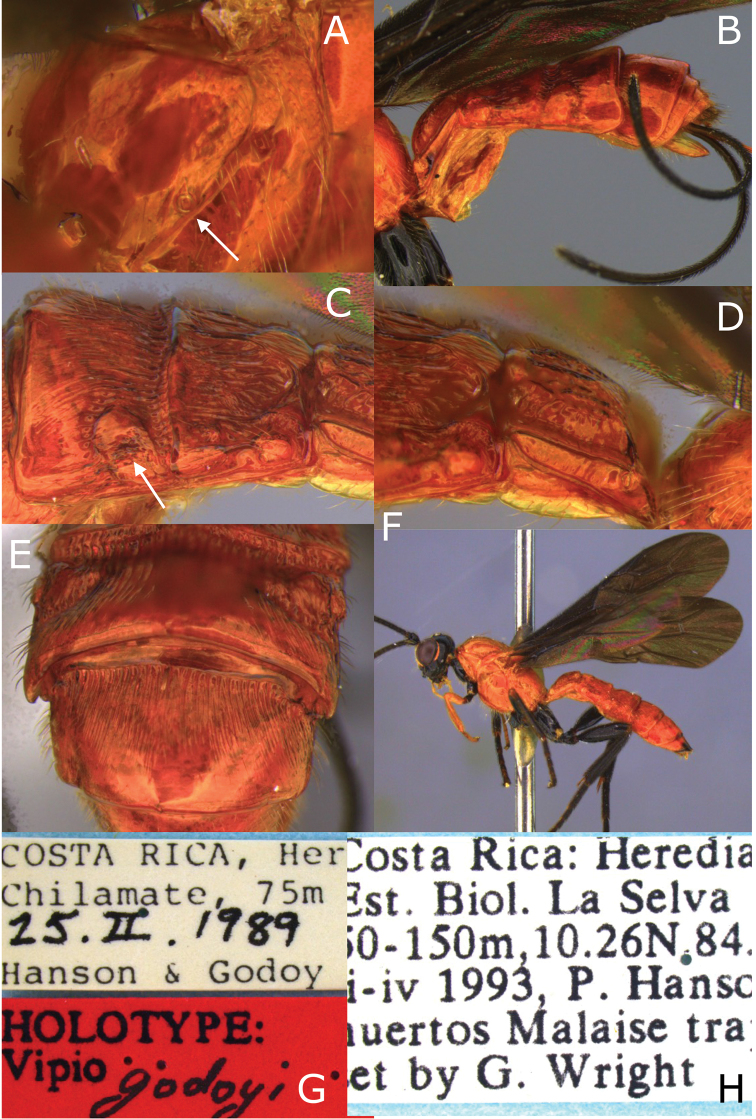
Montaged light micrographs of *Vipio godoyi* sp. nov. **A** Propodeum, oblique dorsal view **B** metasoma, lateral view **C** metasomal tergites II–III, dorso-lateral view **D** metasomal tergite I, dorso-lateral view **E** metasomal tergites III–IV, postero-dorsal view **F** male paratype, lateral habitus **G** holotype labels **H** labels.

### Vipio hansoni sp. nov.

Taxon classificationAnimaliaHymenopteraBraconidae

3A6D0858-C4FE-5BBC-ADAB-5A63F6171D46

http://zoobank.org/E83AADC3-672A-4A18-A82D-1406A0959557

Figures 11 , 12

#### Type material.

Holotype ♀, **Costa Rica**: Limon, Bribri, 4 km NE, ix.1989 (Paul Hanson) (ESUW). Paratypes: **Costa Rica**: 1 ♂, Alajuela, Sta. Clara de San Carlos, 400’, 17.ii.1964 (H.E. Evans) (MCZC); 1 ♂, Heredia, Chilamate, 75 m, 25.iii.1989, (Hanson & Godoy) (ESUW); 1 ♀, Heredia, F. La Selva, 3 km S. Pto. Viejo, 10°26'N, 84°01'W, 31.iii.1980 (H.A. Hespenheide) (ESUW); 1 ♀, same locality, ii-iv.1993 (P. Hanson), huertos Malaise trap set by G. Wright (ESUW).

#### Diagnosis.

*Vipio hansoni* sp. nov. can be recognised by the combination of the predominantly reddish yellow colour, claw with rounded basal lobe, and the presence of two anterior carinae on raised median area of first metasomal tergite.

#### Description.

Holotype ♀ length of body 5.5 mm, of fore wing 5.5 mm and of ovipositor 2.9 mm.

***Head.*** Antenna stout, incomplete with 30 flagellomeres remaining; first flagellomere 4.0 × longer than wide; second flagellomere 3.0 × longer than wide; median flagellomeres 1.15–1.2 × longer than wide; first flagellomere 1.5 longer than second; head transverse; face slightly rugulose; clypeus rugulose; clypeal guard setae typical; HL/HH 0.78; HW/HH 0.87; FH/FW 0.69; EH/HH 0.7; EH/FW 1.15; EW/EH 0.8; ITD 1.65 × TOD; MS 0.23 × EH; LMC 0.3 × HH; third segment of maxillary palpus 4.0 × longer than wide.

***Mesosoma.*** Length of mesosoma 1.74 × height; smooth and shiny; notauli smooth; propodeum mostly smooth except slightly rugose posteromedially.

***Wings.*** Fore wing: length of fore wing 1.0 × body length. PL/LRC 0.87; PW/PL 0.18; length of vein 3RSb 0.88 × combined length of r-rs and 3RSa; length of vein 1M 0.61 × length of (RS+M)a; vein 3RSa reaching anterior wing margin 0.67 × distance between apex of pterostigma and wing tip. Hind wings: uniformly setose; apex of vein C+SC+R with one basal hamule.

***Legs.*** Claw with small, rounded basal lobe.

***Metasoma.*** First tergite 1.1 × longer than posteriorly wide, raised median area oval, rugulose, anteriorly with two carinae joining posteriorly and becoming a single median longitudinal carina reaching apex of disc; surrounding area with short transverse carinae; dorso-lateral carina present, area below rugulose; T II 1.7 × wider than long, longitudinally striate, basal areas smooth and shiny, oblique furrow impressed, striate; T III 1.15 × wider than medially long, longitudinally striate, baso-lateral areas smooth and shiny for the most part; T IV longitudinally striate, T V–VII smooth and shiny; hypopygium ending at the apex of metasoma; ovipositor 0.53 × body length.

***Colour.*** Reddish yellow except head, legs, propleuron, and ovipositor sheath black. Wings dark brown.

#### Variation.

Paratype males (n = 2) as in female, except body length 3.3–3.5 mm; antenna with 32 flagellomeres, gradually shortening and widening distally becoming slightly clavate beyond 25^th^; a small and short median longitudinal carina present on face below antennae; HL/HH 0.88–0.91; EH/HH 0.74–0.76; EH/FW 1.24–1.27; EW/EH 0.72; ITD 2.8–3.1 × TOD; MS 0.16–0.20 × EH; T II–V densely longitudinally striate; fore wing length equal to body length; face yellow with a median black spot above clypeus, third segment of maxillary palpus, antenna basally, fore and middle legs yellow. Paratype females (*N* = 2) with terminal flagellomere acutely pointed.

#### Host.

Unknown.

#### Distribution and seasonality.

So far recorded only from Limon, Alajuela, and Heredia Provinces in Costa Rica. Specimens were collected in March, April, and September.

#### Remarks.

This species is closely related to *V. godoyi* the explanation given under *godoyi* distinguishes both species. This species also is similar to *V. lavignei* sp. nov., but the comments given under *lavignei* separate these species.

#### Etymology.

*Vipio hansoni* is named after Professor Paul Hanson, of the Universidad de Costa Rica, who collected the holotype specimen.

**Figure 11.**
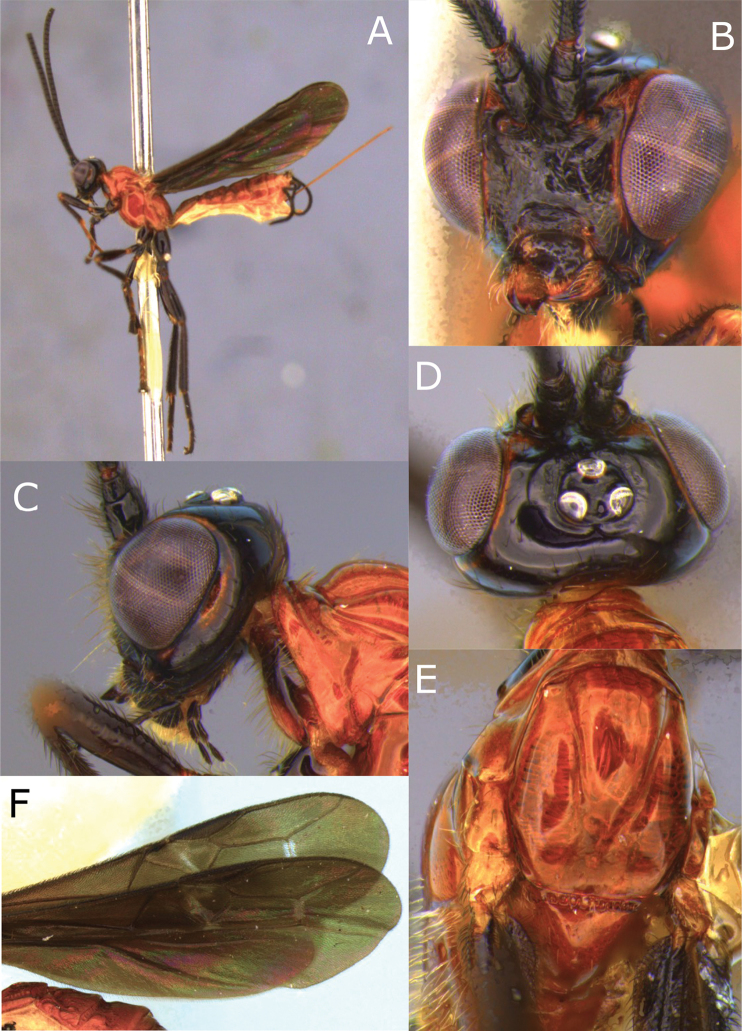
Montaged light micrographs of *Vipio hansoni* sp. nov. **A** Female habitus, lateral view **B** face **C** head and anterior mesosoma, lateral view **D** head, dorsal view **E** anterior mesosoma, dorsal view **F** wings, **G** claw.

**Figure 12.**
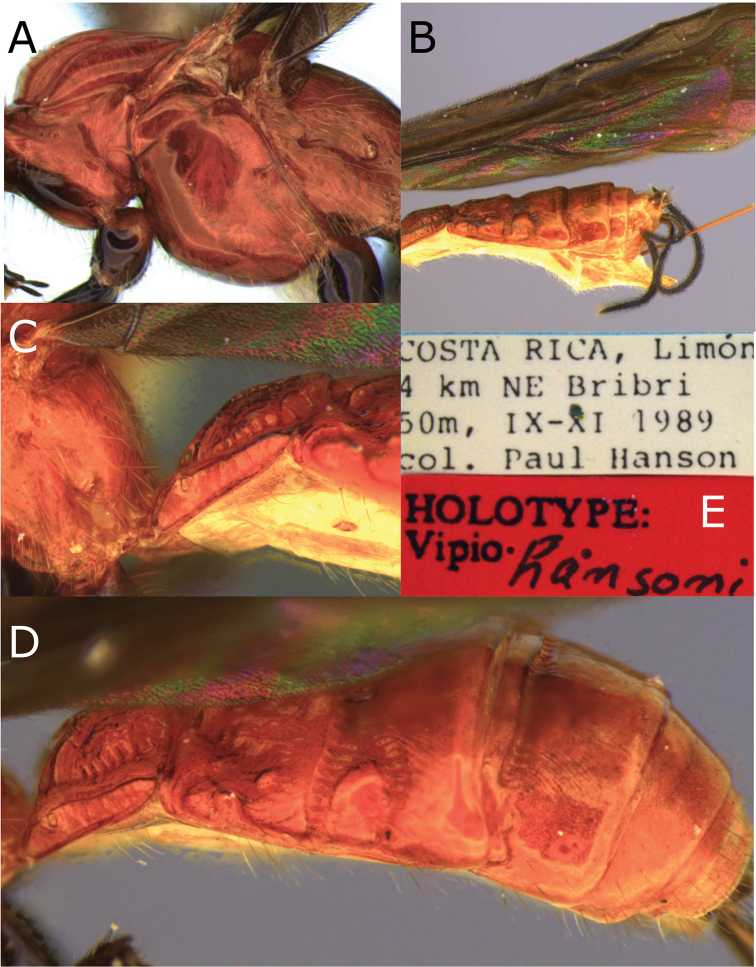
Montaged light micrographs of *Vipio hansoni* sp. nov. **A** Mesosoma, lateral view **B** wings and metasoma, lateral view **C** propodeum and metasomal tergite I, lateral view **D** metasoma, oblique dorsal view **E** holotype labels.

### Vipio lavignei sp. nov.

Taxon classificationAnimaliaHymenopteraBraconidae

8DC8EA29-FB49-5DD0-95E5-ED3171CC395F

http://zoobank.org/C069146D-118E-4BB8-BA8C-DA4CCBB12299

Figures 13 , 14

#### Type material.

Holotype ♀, **Peru**: Tingo Maria 620, 5–12.x.1964 (C.C. Porter) (MCZC). Paratypes: **Peru**: 1 ♀, Tingo Maria, 20–27.i.1968 (A. Garacia and C. Porter) (USNM). **Argentina**: 1 ♂, Tucuman, Horco, Molle, 18–21.iii.1968 (C.C. Porter) (MCZC); 1 ♂, Tucuman, Orán Abra, Grande, 18.iv–5.v.1969, (C.C. Porter) (MCZC).

#### Diagnosis.

*Vipio lavignei* can be recognised by the black head and pronotum, rugo-punctate face, and rectangular, dorso-laterally carinate raised median area of first metasomal tergite.

#### Description.

Holotype and paratype ♀, length of body 9.0–9.2 mm, of fore wing 8.2–8.8 mm, of ovipositor (part exserted beyond apex of abdomen) 3.3 mm, and of antenna 8.8 mm.

***Head.*** Antenna as long as body, with 49 flagellomeres, median flagellomeres 1.2–1.3 × longer than wide, tapering distally; first flagellomere 4.0 × longer than wide, and 1.2 × longer than second, the latter 3.0 × longer than wide; head sub-transverse; clypeus rugose, carinate dorsally; guard setae typical; face rugo-punctate with a small short ridge below antennae; frons rugulose; remainder of head smooth and shiny; HL 0.85 × HH; HW/HH 0.93; FH/FW 0.64; EH/HH 0.6; EH/FW 0.99. EW/EH 0.8; ITD 1.25 × TOD; MS 0.47–0.5 × EH; LMC 0.3 × HH; third segment of maxillary palpus 4.0 × longer than wide.

***Mesosoma.*** Length of mesosoma 1.8 × height. smooth and shiny, except pronotal furrow, crenulate dorsally; notauli smooth; propodeum smooth and shiny.

***Wings.*** Fore wing: length of fore wing 0.96 × body length; PW/PL 0.28; PL/LRC 0.71; length of vein 3RSb 1.1 × combined length of r-rs and 3RSa; length of vein 1M 0.82 × length of (RS+M)a; vein 3RSa reaching anterior wing margin 0.74 × distance between apex of pterostigma and wing tip. Hind wing: uniformly setose; apex of vein C+SC+R with one basal hamule.

***Legs.*** Claw without pointed basal lobe;

***Metasoma.*** First metasomal tergite 1.14 × longer than wide; raised median area rectangular with dorso-lateral carina; slightly rugulose, surrounding area with widely spaced transverse carinae, dorso-lateral carina laminate, area below smooth and shiny; T II 1.8 × wider than long, sparsely longitudinally striate; basal areas smooth and shiny; OF wide, deep, and striate; posterior of tergum smooth; second metasomal suture wide, striate; T III 1.9 × wider than medially long, smooth and shiny posteriorly, baso-lateral areas smooth and shiny for the most part with surrounding area strongly striate; T IV smooth and shiny, except for crenulate transverse basal groove; remainder of metasoma smooth and shiny; hypopygium short, ending at apex of metasoma; ovipositor 0.37 × body length.

***Colour.*** Head, including antenna and palpi, prothorax, mesonotum and legs black; a narrow strip surrounding the eye and a small spot behind each eye yellow; metasomal T I–III red with black tinge; T IV–VII reddish black. Wings smoky;

#### Variation.

Paratype female as in holotype except, length of body 9.2 mm, of fore wing 8.2 mm; face strongly rugose; EH/FW 0.70; HW/HH 0.91; ITD 1.3 × TOD; mesosoma 1.64 × longer than high; length of fore wing 0.88 × body; PL/LRC 0.94; PW/PL 0.24; length of vein 1M 0.75 × length of (RS+M)a; length of vein 3RSb 1.1 × combined length of r-rs and 3RSa; 3RSa reaching anterior wing margin between apex of pterostigma and wing apex at distance 0.67; T I 1.23 × longer than wide; T II 2.1 × wider than long; T III 2.3 × wider than medially long; yellow spot behind antenna absent;. Paratype males (*N* = 2) as in female, except length of body 7.2 mm; length of fore wing 0.89 × body length; antenna 1.0–1.1 × body length; with 38 or 39 flagellomeres; HL 0.89–0.90 × HH; FH/FW 0.67; EH/HH 0.62–0.64; EW/EH 0.79; EH/FW 0.95; ITD 1.7 × TOD; MS 0.39 × EH; T II–IV uniformly longitudinally striate.

#### Distribution and seasonality.

Argentina and Peru. Two specimens from Argentina were collected in March and May and two from Peru in October.

#### Etymology.

Named after UW Professor Emeritus Robert J. Lavigne, in honour of his diverse contributions to entomological research and his role in promoting the insect systematics program at the University of Wyoming.

#### Remarks.

This species is apparently closely related to *V. hansoni* sp. nov. because of its strongly sclerotised metasoma, and wide, deep 2^nd^ metasomal suture. *V. lavignei* can be separated by the rugo-punctate face (smooth and shiny in *hansoni* sp. nov.), the black mesonotum (yellow in *hansoni*), and the rectangular T I raised median area (oval in *hansoni*).

**Figure 13.**
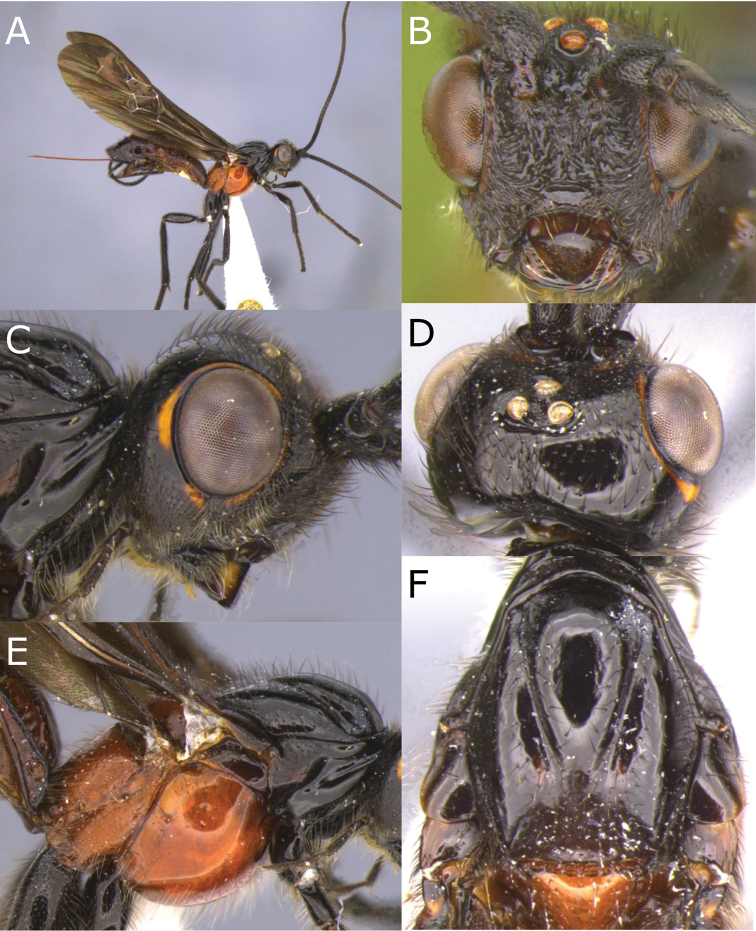
Montaged light micrographs of *Vipio lavignei* sp. nov. **A** Female habitus, lateral view **B** face **C** head and anterior mesosoma, lateral view **D** head, near dorsal view **E** mesosoma and metasomal tergite I, lateral view **F** mesoscutum and scutellum, dorsal view.

**Figure 14.**
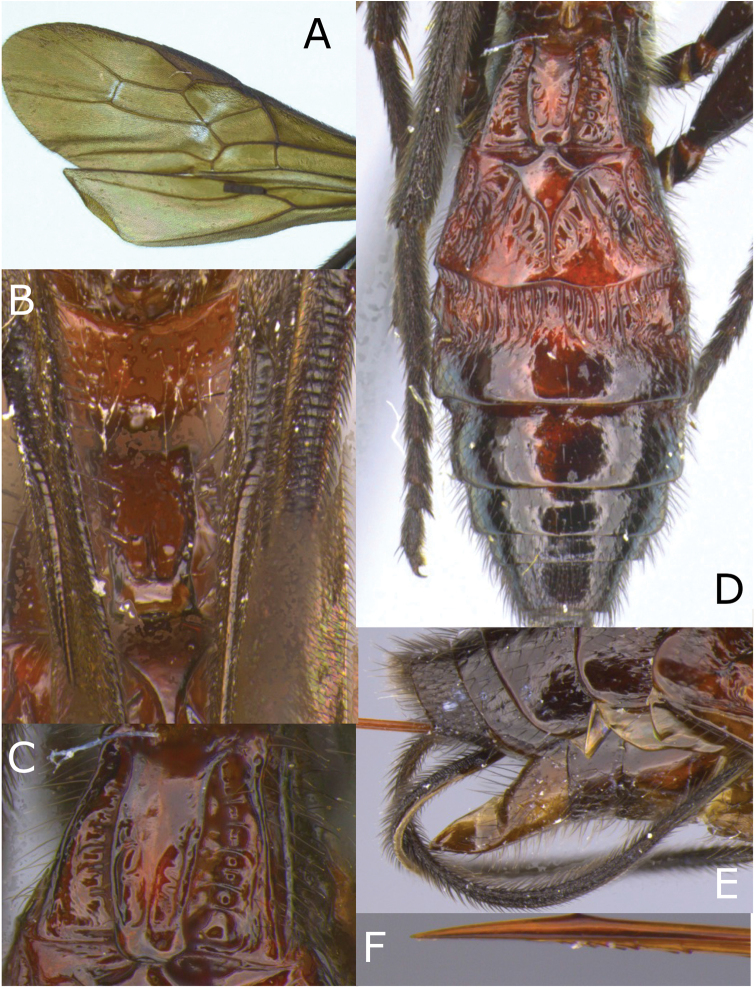
Montaged light micrographs of *Vipio lavignei* sp. nov. **A** Wings **B** propodeum and metasomal tergite I, dorsal view **C** metasomal tergite I, near dorsal view **D** metasoma, dorsal view **E** apex of metasoma, lateral view **F** apex of ovipositor, lateral view.

### Vipio melanocephalus

Taxon classificationAnimaliaHymenopteraBraconidae

Brullé, 1846

EB8DE0E6-68AA-5C4D-99A1-2CA1057A9C0C

Figures 15 , 16

Vipio melanocephalus Brullé, 1846: 445; Shenefelt, 1978: 1853.

#### Type material.

Holotype ♀, *Vipio melanocephalus* Brullé, 1846, **Brazil**: del Rio-Grande (no other data) (MNHN).

#### Additional specimens examined.

**Bolivia**:1 ♀, 3 ♂, Sara (no date) (Steinbach) (MCZC).

#### Diagnosis.

*Vipio melanocephalus* can be recognised by the combination of its size (body length > 1cm), ovipositor length (≥ 2 × body length), largely black head and claw with large, acutely pointed basal lobe.

#### Description.

Females, length of body 10.8–11.5 mm, of fore wing 7.1–7.3 mm, and of ovipositor (part exserted beyond apex of abdomen) 21.0–27.0 mm.

***Head.*** Antenna stout; first flagellomere 2.7 × longer than wide, 1.6 × longer than second, the latter 1.4 × longer than wide; (data could not be recorded for other antennal characters because the only available specimen with antenna was dirty and broken); head transverse; clypeal guard setae typical; face slightly rugulose laterally; remainder of head smooth and shiny; HL 0.72–0.74 × HH; HW/HH 0.77–0.79; EH/HH 0.59–0.61; EH/FW 0.81; EH/HH; EW/EH 0.81; 0.59–0.60; ITD 1.65 × TOD; MS 0.39–0.40 × EH; LMC 0.4 × HH; third segment of maxillary palp 4 × wider than long.

***Mesosoma.*** Length of mesosoma 1.79–1.81 × height; smooth and shiny, except pronotum rugulose dorso-laterally; notauli smooth; propodeum rugose medially, slightly rugulose laterally, smooth or punctate on basal and lateral margins.

***Wings.*** Fore wing: length of fore wing 0.61–0.67 × body length; PL/LRC 0.75–0.80; length of vein 1M 0.69–0.73 × length of (RS+M)a; length of vein 3RSb 0.83–0.90 × combined length of r-rs and 3RSa; vein 3RSa reaching anterior wing margin between apex of pterostigma and wing apex at distance 0.5–0.53. Hind wing: with glabrous area basally; with one basal hamule.

***Legs.*** Claw with pointed basal lobe.

***Metasoma.*** First tergite 1.4–1.42 × longer than wide, raised median area oval, rugulose, with smooth anterior area continuing posteriorly as median longitudinal carina, surrounding area with transverse carinae, dorso-lateral carina present; T II –IV longitudinally striate; T II 1.1 × wider than long, basal areas smooth, medio-basal area continuing posteriorly as a median longitudinal carina, oblique furrows strongly impressed; T III 1.2 × wider than medially long, baso-lateral areas present; remainder of metasoma smooth and shiny; hypopygium extending 1.0 mm beyond apex of metasoma; ovipositor 2.0–2.3 × body length.

***Colour.*** Predominantly orange; head largely black, face sometimes orange; frons sometimes yellow laterally; antenna rufous or black; labial and maxillary palp reddish black; legs black or reddish black, except fore legs beyond trochanter and middle tarsi orange.

#### Variation.

Paratype males (*N* = 3) as in female, except length of body 6.5 mm; median flagellomeres longer than wide (antenna broken beyond F43); HL 0.86–0.88 × HH; EH/HH 0.72–0.73; EW/EH 0.80; EH/FW 1.03–1.06; FH/FW 0.5–0.52; ITD 2.5 × TOD; MS 0.18 × EH; punctation on pronotal furrow sometimes extends laterally; T V striate; face yellowish or reddish white with a reddish brown triangular spot above clypeus; frons yellow, remainder of head black; legs yellow to black;.

#### Remarks.

This species is similar to *V. fiebrigi* Bréthes, but can be readily separated by the characters discussed under *V. fiebrigi*.

**Figure 15.**
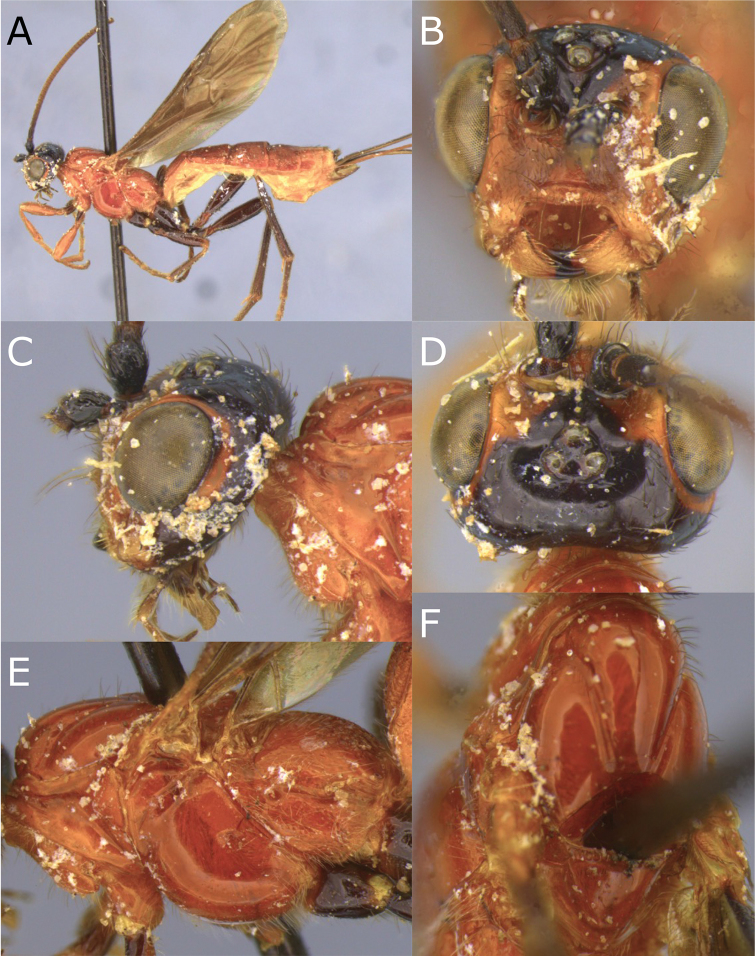
Montaged light micrographs of *Vipio melanocephalus*. **A** Female habitus, lateral view **B** face **C** head and anterior mesosoma, lateral view **D** head, dorsal view **E** mesosoma, lateral view **F** anterior mesosoma, dorsal view.

**Figure 16.**
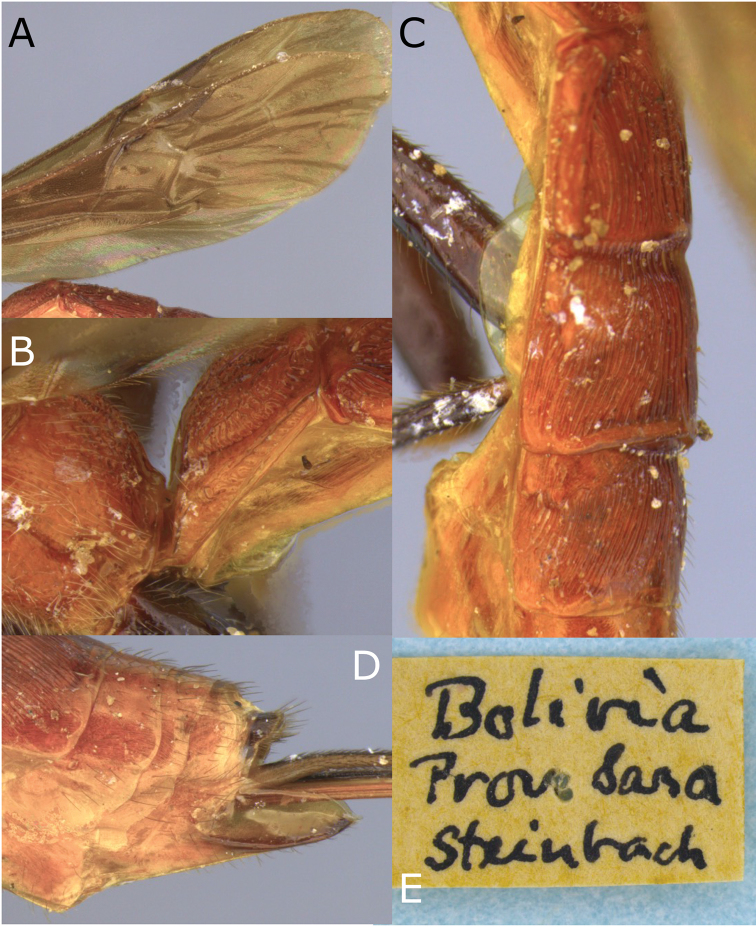
Montaged light micrographs of *Vipio melanocephalus*. **A** Wings **B** propodeum and metasomal tergite I, near dorsal view **C** metasomal tergites II–V, lateral view **D** apex of metasoma and hypopygium, lateral view **E** data label.

### Vipio paraguayensis

Taxon classificationAnimaliaHymenopteraBraconidae

Szépligeti, 1906

9DBCE4D9-6B47-5F39-BF77-720E0630BD46

**Figure 17.**
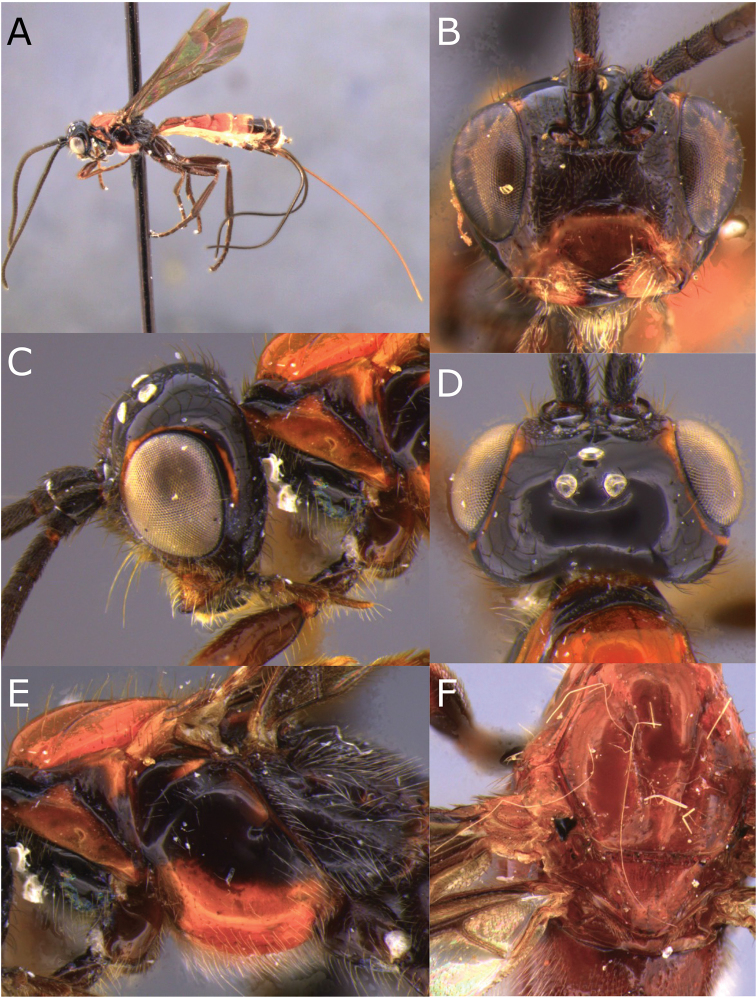
Montaged light micrographs of *Vipio paraguayensis*. **A** Female habitus, lateral view **B** face **C** head and anterior mesosoma, dorso-lateral view **D** head, dorsal view **E** mesosoma, lateral view **F** mesoscutum and scutellum, dorsal view.

**Figure 18.**
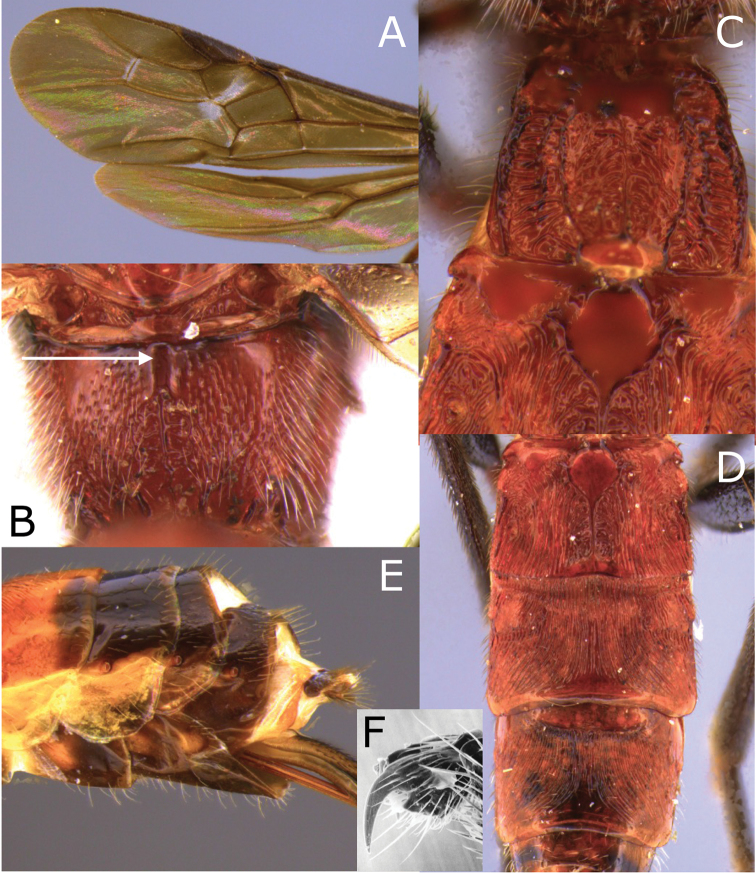
Montaged light and scanning electron micrographs of *Vipio paraguayensis*. **A** Wings **B** propodeum **C** metasomal tergites I and II, dorsal view **D** metasomal tergites II–IV, dorsal view **E** apex of metasoma and hypopygium, lateral view **F** SEM of claw.

**Figure 19.**
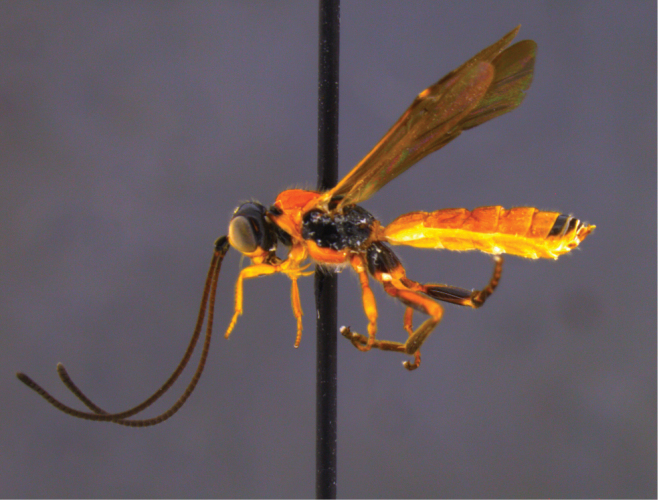
*Vipio paraguayensis* male, lateral habitus.

Figures 17 , 18 , 19

Vipio paraguayensis Szépligeti, 1906: 157; Shenefelt, 1978: 1857.

#### Type material.

Holotype ♀, *Vipio paraguayensis*Szépligeti 1906, **Paraguay**, Villa Encarnacion, 7.xii.1904 (Schrottky) (HNHM type No. 832).

#### Additional material examined.

**Argentina**: 1 ♀, Buenos Aires, 1.i.1950 (J. Foerster) (USNM); 1 ♀, Buenos Aires, San Clement del Tuyu, xi.1950 (J. Foerster) (CNCI); 1 female, Pronunciamiento Entre Rios, ii.1965 (CNCI); 1 ♀, Tucuman, Va. Padro Monte-R. Nio, 25.iv.1966 (C.C. Porter) (USNM); 1 ♀, La Plata, Fac., Agronomia, 22.xii.1968 (C.C. Porter) (USNM). **Bolivia**: 3 ♀♀, Corolco (HNHM); 1 ♂, Corolco, 1800 m, 3–8.xii.1955 (L.E. Pena) (CNCI). **Brazil**: 3 ♀♀, Nova Teutonia 27°11'S, 52°23'W 300–500 m, vii-xi.1968 (F. Plaumann) (CNCI). **Chile**: 1 ♀, Conesa, Rio Negro, i.1954 (F.H. Waltz) (USNM). **Colombia**: 1 ♀, Cundinamarca Monterredondo, 10.xii.1958 (J. Foerster) (USNM). **Trinidad**: 1 ♂, Port of Spain (W.S. Brooks); 1 ♀, “1–9” Maracas, xii.1977, malaise trap (CNCI); 3 ♀, Curepe, 10.iii.1978, 28.iii.1978, 6.xii.1967; 1 ♀, San Andrew, nr. Valencia 23.iii.1985 (G.F. & J.F. Hevel) (CNCI); 1 ♀, Cocos Bay, 28–29.vi.1982 (J.M. Carpenter & J.S. Edgerly) (USNM); 1 ♀, Caranege, 14.x.1918 (Harold & Morrison) (USNM); 1 ♀, St. Augustine, 2.iii.1953 (F.J. Simmonds) (USNM); 1 ♂, Aripo Savana, 26.x.1918 (Harold & Morrison) (USNM); 2 ♂, Aripo Cumuto (R. Thaxter) (USNM). **Venezuela**: 1 ♂, El Tucuco, 200 m 19.iv.1981 (L. Masner) (USNM).

#### Diagnosis.

May be distinguished from other Neotropical *Vipio* species by the combination of long ovipositor (1.5–1.9 × body length), presence of an acutely pointed basal lobe to claw and a short mid-anterior, rather wide, carina on the propodeum.

#### Description.

**Females**, length of body 5.6–8.4 mm, of fore wing 4.6–6.8 mm, of ovipositor (part exserted beyond apex of abdomen) 6.4–10.2 mm and of antenna 4.5–7.0 mm.

***Head.*** Antenna robust, 0.85–0.87 × body length, with 42–48 flagellomeres; first flagellomere 1.5–1.6 × longer than second, 2.0 × longer than wide; second flagellomere 1.6 × longer than wide; median flagellomeres quadrate; distal flagellomeres wider than long, except terminal flagellomere longer than wide, with apex bluntly rounded; head sub-transverse; face uniformly punctate, rarely rugulose laterally, remainder of head smooth and shiny; clypeus higher in profile, slightly rugulose, clypeal guard setae typical; HL 0.8–0.87 × HH; HW/HH 0.87–0.9; FH/FW 0.47–0.49; EH/HH 0.67–0.70; EH/FW 0.70–0.94; EW/EH 0.78–0.8; ITD 1.7 × TOD; MS 0.3–0.35 × EH; LMC 0.3 × HH; third segment of maxillary palpus 4.0 × longer than wide.

***Mesosoma.*** Length of mesosoma 1.78–1.8 × height; pronotum smooth and shiny, except at furrow, punctate dorso-laterally; notauli smooth; propodeum smooth and punctate laterally with a shallow median furrow, having a basally smooth median longitudinal carina.

***Wings.*** Fore wing: length of fore wing 0.75–0.80 × body length; PL/LRC 0.92–0.94, PW/PL 0.24–0.27; length of vein 3RSb 0.91–0.95 × combined length of r-rs and 3RSa; length of vein 1M 0.62–0.64 × length of (RS+M)a; 3RSa reaching anterior wing margin between apex of pterostigma and wing apex at distance 0.53–0.57. Hind wing: uniformly setose or with sparse setosity basally; apex of C+SC+R with one basal hamule.

***Legs.*** Claw with pointed basal lobe.

***Metasoma.*** T I 1.34–1.38 × longer than wide, raised median area oval, anterior smooth area narrowing posteriorly, becoming a median longitudinal carina with short transverse carinae posteriorly; carinate at lateral margin; surrounding area with short transverse striae; dorso-lateral carina present, area below crenulate; T II 1.35–1.50 × wider than long, baso-lateral areas smooth and triangular; baso-medial area becoming a median longitudinal carina posteriorly and reaching a small raised smooth area at the apex of tergum; remainder of the tergum longitudinally striate, oblique furrows impressed, striate; T III 1.3–1.7 × wider than medially long longitudinally striate, baso-lateral areas distinct; T IV longitudinally striate with small baso-lateral area; T V–VII smooth and shiny; hypopygium extending 0.4–0.7 mm beyond apex of metasoma; ovipositor 1.1–1.4 × body length.

***Colour.*** Black and reddish yellow; face black or reddish black; base of mandible, and a narrow strip around eyes yellow; remainder of head black; pronotum dorsally (sometimes), propleuron (sometimes), mesopleuron, scutellum (except edges), propodeum, metapleuron, legs, metasomal T V–VII, ovipositor sheath black; remainder of body reddish yellow. Wings smoky, pterostigma yellowish brown.

**Male.** As in female, except length of body 4.3–6.4 mm, of fore wing 0.89–0.94 × body length; antenna with 36–46 flagellomeres, all flagellomeres longer than wide, except distal 5 or 6 which gradually become clavate (Fig. 19); HL 0.83–0.87 × HH; EH/HH 0.88–0.90; EH/FW 0.85–0.87; FH/FW 0.56–0.59; ITD 3.0 × TOD; MS 0.14–0.16 × EH; EW/EH 0.61; face smooth and shiny, yellowish white with a black spot above clypeus; segments 2 and 3 of maxillary palp distinctly expanded.

#### Remarks.

*Vipio paraguayensis* can be easily recognised by the combination of the presence of a pointed basal lobe on the claw, the presence of a median longitudinal carina on the propodeum, the densely striate T II–IV, and the long ovipositor. Based on the presence of the median longitudinal carina on propodeum, this species may be closely related to *V. boliviensis* sp. nov. However, the presence of a pointed basal lobe on the claw, longitudinal striations on the metasoma, and longer hypopygium in *paraguayensis* separate it from *boliviensis* sp. nov. (in which the basal lobe of the claw is rounded, T III and IV are transversely striated, and the hypopygium is short). Males of this species can be confused with males of *V. belfragei* because of the expanded third and fourth maxillary segments, but the clavate antenna in *paraguayensis* (as opposed to a filiform antenna in *belfragei*) readily separate these two species. Another useful character is the presence of a median longitudinal carina on the propodeum in this species, as opposed to several short carinae posteriorly in *belfragei*.

### Vipio porteri

Taxon classificationAnimaliaHymenopteraBraconidae

Inayatullah, Sabahatullah & Ain Tahira, 2015

7A733001-3F69-5E12-A865-B05AF9BEA93A

Figure 20

Vipio porteri Inayatullah, Sabahatullah & Ain Tahira, 2015: 132–133, fig. 4 (note that the SEM figures published therein are distorted by approximately 1.3:1.0).

#### Type material.

Holotype ♀, **Argentina**, Tucuman, Las Cejas, 8.iii–11.iv.1968 (C.C. Porter) (MCZC). Paratypes: **Argentina**: 9 ♀, 2 ♂, Tucuman, Las Cejas, 8.iii–11.iv.1968 (C.C. Porter) (MCZC); 3 ♀, 2 ♂, 22.ii–13.iii.1968 (C.C. Porter) (MCZC); 1♀, 1♂, same data, except 21.i–21.ii, 1968; 1 ♂, same data, except 1–21.i.1968; 1 ♀, same data, except 2.iv.1966; 3 ♀, near Las Cejas, 20.iv.1968 (C.C. Porter) (MCZC); 2 ♀, 5 ♂, Las Cejas, 11 km W, 1–16.xi.1967 (C.C. Porter) (MCZC); 1 ♀, same data, except 1.xi.1967; 2 ♀, same data, except 18.xi–4.xii.1967; 1 ♀, same data, except 27.v–14.viii.1968; 1 ♀, same data, except 24.ix–17.x.1968; 1 ♀, same data, except xii.1967; 4 ♀, 1 ♂, same data, except 24.ix–17.x.1968 (ESUW); 1 ♀, Las Cejas, 11 km. W, 12.iv–5.v.1968 (L. Stange) (EMUS).

#### Distribution and seasonality.

Known only from Argentina. Specimens collected between September and May.

**Figure 20.**
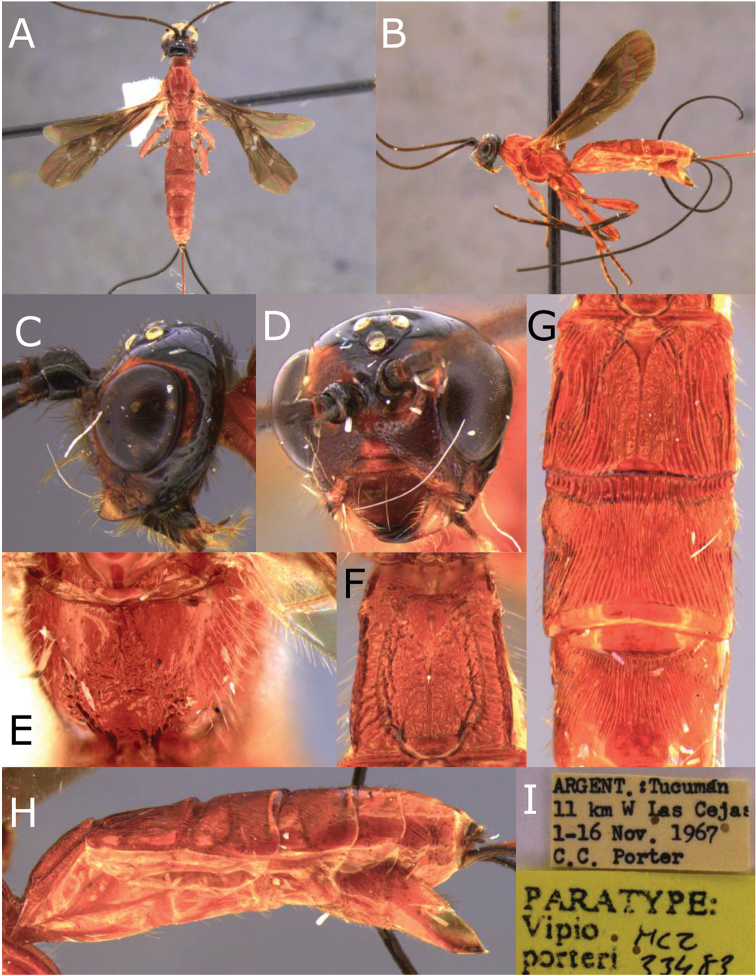
Montaged light micrographs of *Vipio porteri* paratype female. **A** Habitus, dorsal view **B** habitus, lateral view **C** head, lateral view **D** face **E** propodeum **F** metasomal tergite I **G** metasomal tergites II–IV, dorsal view **H** metasoma lateral view **I** labels.

### Vipio quadrirugulosus

Taxon classificationAnimaliaHymenopteraBraconidae

(Enderlein)

E6839086-0CD8-5A92-9F7B-34C4DB33E119

Figures 21 , 22

Craspedolcus quadrirugulosus Enderlein, 1920: 94; Shenefelt, 1978: 1673; Isomecus quadrirugulosus: Quicke & van Achterberg, 1990: 253, 256; Vipio quadrirugulosusYu et al., 2016.

#### Type material.

Holotype♀, *Craspedolcus quadrirugulosus* Enderlein, 1920, **Ecuador**, Bucay, (no additional data) (APSW).

#### Additional material examined.

**Costa Rica**: 1♀, Alajuela, Rio-Laguna de Arenal, 500 m, 14.iii.1988 (P. Hanson) (RMSEL); 1 ♀, Guanacaste, Hacind, La Pacifica, Canas, 3 km N, 24.i.1972 (G. Frankie) (TAMU). **El Salvador**: 1 ♀, Quezaltepeque, 20.vi.1961 (M.E. Irwin) (USNM). **Guatamala**: 1 ♀, Yepocopa, v.1948, (H.T. Dalmat) (USNM). **Honduras**: 1 ♀, Mt. Pine Ridge, 2–6.vii.1967 (Porter) (USNM). **Mexico**: 2 ♀, Chiapas, Pichucalco, 11.6 mi. SE, 3.viii.1980, (Schaffner, Weaver, Freidlander) (TAMU); 1 ♀, Chiapas, Huixtla, 20 mi. N, 3000’, 1.vi.1969 (W.R.M. Mason) (TAMU); 1 ♀, Chiapas, Pichucalco, 9.5 mi. NW, 3.viii.1980 (TAMU); 1 ♀, Chiapas, Campostela Rio de Marcos, 42.7 mi. SW, 100’, 1.i.1942, (R.R & H.E. Murray) (TAMU). 1 ♀, Chiapas, No 2154 (C.F. Baker), 1 ♀, Morelos, Cuernavaca, iii.1945 (N.L.H. Krauss) (USNM); 1 ♀, Tabasco, Cardina, 8.ix.1974, (G. Bohart & W. Hanson) (USU). **Panama**: 1 ♀, Canal Zone, Albrook Field, 25.x.1937 (USNM); 1 ♀, Panama City, Bella Vista, 7.viii.1924, (N. Banks) (USNM); 1 ♀, Canal Zone, Ft. Clayton, xii.1946 (N.L.H. Krauss) (USNM).

#### Diagnosis.

This species can be easily recognised from all other species by the black metasoma. Additionally, T II–V are densely striate longitudinally and the claws have a pointed basal lobe.

#### Description.

**Females** (*N* = 17) length of body 4.8–9.3 mm; of fore wing 5.3–8.3 mm, of ovipositor 2.3–2.6 mm, and of antenna 4.6–8.5 mm.

***Head.*** Antenna 0.94–1.0 × body length; with 39–47 flagellomeres; first flagellomere 1.4 × longer than second, 2.5 × longer than wide; second flagellomere 2.0 × longer than wide; median flagellomeres 1.0–1.4 × longer than wide; antenna gradually tapering towards apex; terminal flagellomere acutely pointed apically; head transverse; clypeus rugulose; clypeal guard setae consist of one seta above each anterior-tentorial pit; face smooth and shiny or sparsely punctate; remainder of head smooth and shiny; HL/HH 0.76–0.78; HW/HH 0.75–0.78; FH/FW 0.6–0.62; EH/HH 0.63–0.67; EH/FW 0.99–1.1; EW/EH 0.72–0.74; ITD 1.35–1.7 × TOD; MS 0.32–0.35 × EH; LMC 0.3 × HH; third segment of maxillary palpus 4.0 × longer than wide.

***Mesosoma.*** Length of mesosoma 1.5–1.64 × height; smooth and shiny, except dorsally crenulate pronotal furrow; pronotum usually carinate antero-laterally; notauli smooth; propodeum mostly smooth except slight rugose apically.

***Wings.*** Fore wing: length of fore wing 1.0–1.1 × body length; PL/LRC 0.9–1.0; PW/PL 0.19–0.24; length of vein 3RSb 0.77–0.82 × combined length of r-rs and 3RSa; length of vein 1M 0.66–0.71 × length of (RS+M)a; 3RSa reaching wing margin 0.63–0.65 distance between apex of pterostigma and wing apex. Hind wing: basally uniformly setose; apex of vein C+SC+R with one or two basal hamules.

***Legs.*** Claw with wide pointed basal lobe.

***Metasoma.*** First tergite 1.2 × longer than posteriorly wide; raised median area oval, smooth or rugulose anteriorly with or without a complete median longitudinal carina; always with a median longitudinal carina and areolate-rugose posteriorly; surrounding area with transverse carinae; dorso-lateral carina lamelliform; T II–V longitudinally striate; T II 2.0–2.1 × wider than long, basal areas smooth and shiny oblique furrow strongly impressed, striate; T III 2.2–2.5 × wider than medially long, baso-lateral areas usually carinate-rugulose; T IV baso-lateral area rugulose; T V smooth and shiny, rarely striate; remainder of metasoma smooth and shiny; hypopygium short, ending at apex of metasoma; ovipositor 0.35–0.48 × body length.

***Colour.*** Head black, except a yellowish red and/or yellowish stripe surrounding the eye and basal half mandible reddish yellow to yellow; antenna, maxillary and labial palpi, prosternum, propleuron, legs, and metasoma black. Wings brownish black, pterostigma black.

#### Distribution and seasonality.

Ranging from northern Mexico southwards to Ecuador (with records from Costa Rica, El Salvador, Ecuador, Guatemala, Honduras, Mexico and Panama). Specimens from Costa Rica were collected from January through March, June in El Salvador, May in Guatemala, July in Honduras, August through December in Panama, and from June through September in Mexico.

#### Remarks.

*Vipio quadrirugulosus* appears closely related to the Nearctic *V. rugator* because of presence of a raised median area on the face, a strongly sclerotised and densely longitudinally striate metasoma, short ovipositor, and the presence of a pointed basal lobe on the claw. However, the black metasoma and the presence of carinae on the raised median area in *V. quadrirugulosus* will readily separate this species from *V. rugator* in which the metasoma is yellow or reddish yellow and the raised median area of the first tergite is areolate and rugose and lacks such a carina.

**Figure 21.**
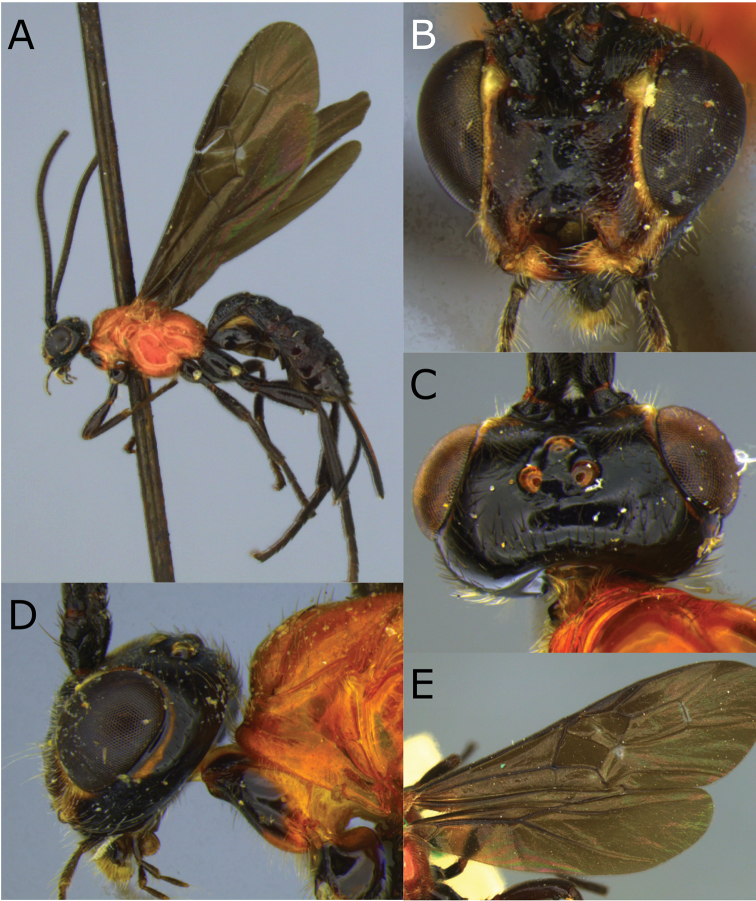
Montaged light micrographs of *Vipio quadrirugulosus*. **A** Female habitus, lateral view **B** face **C** head, dorsal view **D** head and anterior mesosoma, lateral view **E** wings.

**Figure 22.**
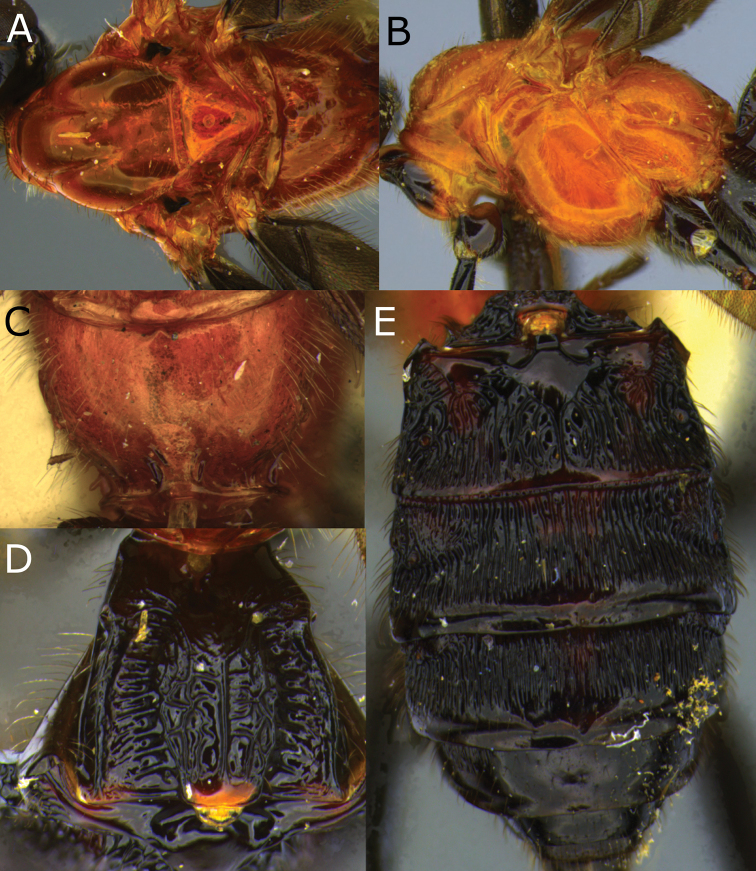
Montaged light micrographs of *Vipio quadrirugulosus*. **A** Mesosoma, dorsal view **B** mesosoma, lateral view **C** propodeum **D** metasomal tergite I, dorsal view **E** metasomal tergites II–VI.

### Vipio strigator

Taxon classificationAnimaliaHymenopteraBraconidae

(Bréthes, 1913)

7EFD4F55-AF3D-5ED0-A524-4B0DB4D8E2E6

Figures 23 , 24

Iphiaulax strigator Bréthes, 1913: 79; Shenefelt, 1978: 1797; Vipio strigator: Quicke & Genise, 1994: 44.

#### Type material.

Holotype, ♀, *Iphiaulax strigator* Bréthes, 1913, **Argentina**: “Potrerillo”, Mendoza, (no date) 4000’ (IFML).

#### Additional material examined.

**Argentina**: 1 ♀, Misiones Panamb, 24.xi.1954 (Monro’s, Willink); 1 ♀, Misiones San Pedro, 15.xi.1973 (Tomsic, Willink); 1 ♀, Misiones Bernardino de Irigoyen 12.xi.1973 (Tomsic, Willink); 1 ♂, Misiones San Pedro, 16.xi.1973 (Willink, Tomsic); 2 ♀, Misiones Iguazo, 30.i–13.iii.1945 (Hayward, Willink, & Golbach) (IFML). **Brazil**: 5 ♀, 11 ♂, Nova Teutonia, 27°11'S, 52°23'W, 300–500 m, i.1965 (F. Plaumann) (CNCI); 16 ♀, same data, except xi.1966; 12 ♀, xi.1968; 3 ♀, xi.1964; 2 ♀, xii.1966; 2 ♀, same data, except 20.xii.1955, 2.xi.1962. **Paraguay**: 1 ♀, Villarrica, ii.1951 (Pfannl) (IFML). **Peru**: 1 ♀, Valle Chanchamayo, 800 m, 13.viii.1951 (Weyrauch) (IFML).

#### Diagnosis.

Ovipositor less than 0.5 × body length, predominantly red, head black; face with raised triangular area; propodeum with raised stub-like area and with four or five carinae postero-medially that usually reach the middle of propodeum; claw with strong pointed basal lobe.

#### Description

(**Females**, *N* = 51). Length of body 6.0–10.1 mm, of fore wing 6.6–11.1 mm, of ovipositor (part exserted beyond apex of abdomen) 2.4–3.2 mm, and of antenna 6.0–9.5 mm.

***Head.*** Antenna 0.74–0.97 × body length, with 43–50 flagellomeres; first flagellomere 2.5 × longer than wide, 1.4 × longer than second, the latter 2.0 × longer than wide; median flagellomeres as quadrate; terminal flagellomere acutely pointed apically; clypeus rugulose, clypeal guard setae typical; face smooth to sparsely punctate, with a raised triangular area above clypeus; remainder of head smooth and shiny; HL 0.76–0.86 × HH; HW/HH 0.69–0.88; FH/FW 0.61–0.69; EH/HH 0.64–0.72; EH/FW 1.0–1.1; EW/EH 0.68–0.7; ITD 1.15–1.3 × TOD; MS 0.36–0.42 × EH; LMC 0.3 × HH; third segment of maxillary palpus 4.0 × longer than wide.

***Mesosoma.*** Length of mesosoma 1.54–1.7 × height; smooth and shiny; propodeum with a raised postero-medially area with 4–5 longitudinal carinae reaching almost the middle of propodeum.

***Wings.*** Fore wing: length of fore wing1.0–1.1 × body length; PW/PL 0.25–0.35; PL/LRC 1.0–1.05; length of vein 3RSb 0.84–0.87 × combined length of r-rs and 3RSa; length of vein 1M 0.6–0.7 × length of (RS+M)a; vein 3RSa reaching wing margin at distance 0.59–0.62 between apex of pterostigma and wing apex. Hind wing: uniformly setose basally; apex of vein C+SC+R with one or two basal hamules.

***Legs.*** Claw with strong pointed basal lobe.

***Metasoma.*** First metasomal tergite 1.1–1.15 × longer than wide, raised median area oval, gradually narrowing posteriorly, pointed anteriorly, carinate rugose, surrounding area with short transverse carinae, dorso-lateral carina lamelliform; T II–V longitudinally striate; T II 2.0–2.25 × wider than long, medio-basal area smooth and shiny, oblique furrows strongly impressed, striate; T III 2.6–2.9 × wider than medially long; baso-lateral areas of T III and IV rugulose; T VI–VIII smooth and shiny; hypopygium short, ending at apex of abdomen; ovipositor 0.3 × body length.

***Colour.*** Largely red; head, including antenna and palpi, black except basal half of mandible reddish yellow and a yellow or yellowish red stripe surrounding the eye; pronotum reddish black with pronotal furrow red; prosternum, propleuron, basal 0.8 of mesopleuron, middle and lateral lobe of mesonotum laterally, scutellum apically, legs, and ovipositor sheath black. Wings brownish-black.

**Male** (*N* = 12). As in female, except length of body 4.6–6.5 mm, fore wing as long as body length; antennae 1.0–1.1 × body length; HL 0.84–0.89 × HH; EH/HH 0.71–0.76; EH/FW 1.34–1.4; FH/FW 0.77–0.82; EW/EH 0.71–0.75; ITD 1.6–1.7 × TOD; MS 0.21–0.27 × EH.

#### Remarks.

*Vipio strigator* can be recognised by the combination of reddish black markings on the mesosoma, the short hypopygium, and the short ovipositor. This species is similar to the Nearctic *V. rugator* because of the presence of a raised area on face, short hypopygium, and short ovipositor in both species. However, the red coloration with reddish black markings on the mesosoma and a carinate propodeum in *strigator* will readily separate it from *rugator* (in which the mesosoma lacks black markings and propodeum lacks such carinae).

**Figure 23.**
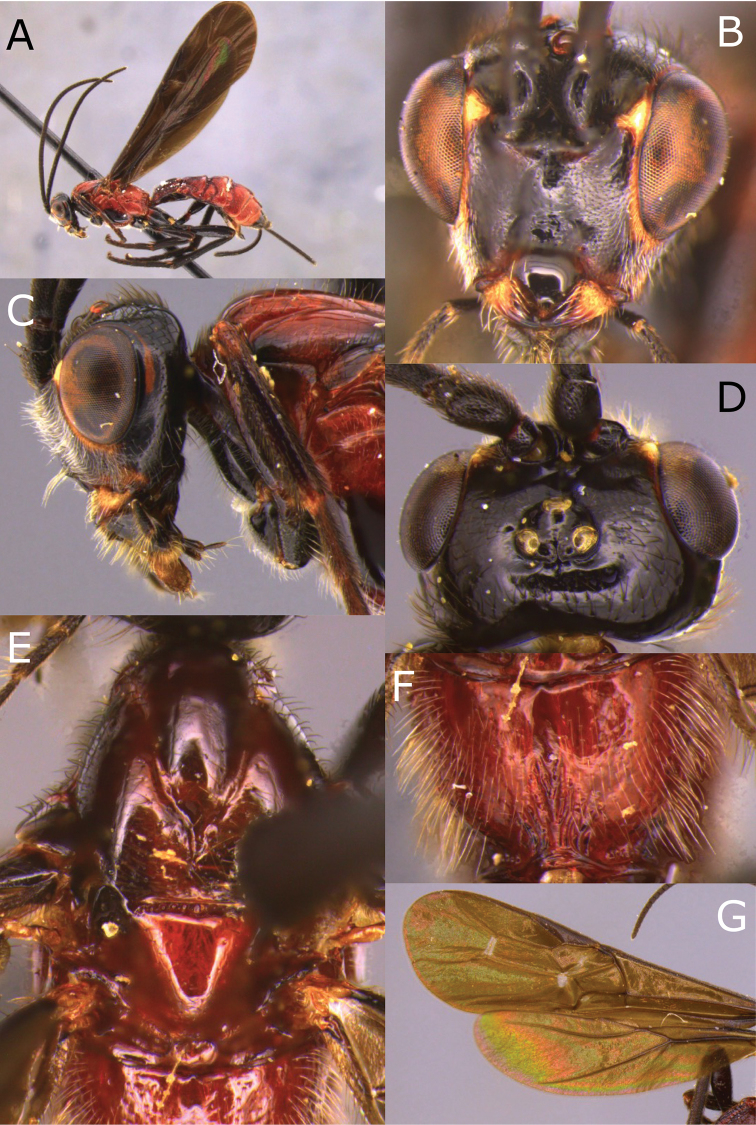
Montaged light micrographs of *Vipio strigator*. **A** Habitus lateral view **B** face **C** head and anterior mesosoma, lateral view **D** head, dorsal view **E** mesoscutum and scutellum, dorsal view **F** propodeum **G** wings.

**Figure 24.**
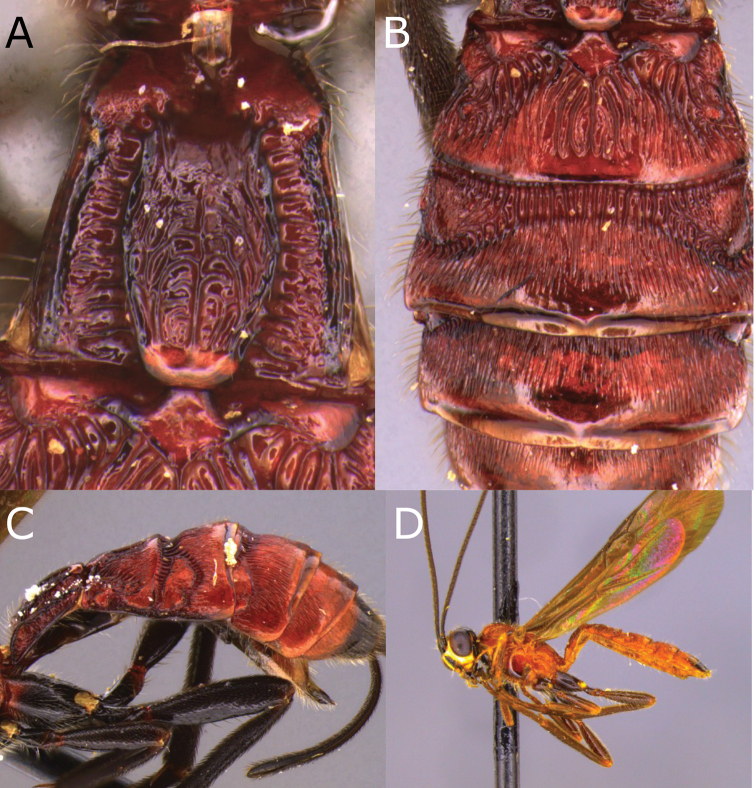
Montaged light micrographs of *Vipio strigator*. **A** Metasomal tergite I, dorsal view **B** holotype, metasomal tergites II–V **C** metasoma lateral view **D** male habitus, lateral view.

### Vipio thoracica

Taxon classificationAnimaliaHymenopteraBraconidae

(Ashmead), 1900

E3072DFF-DBC4-515D-863D-3BE83F4D790D

Figures 25 , 26

Glyptomorpha thoracica Ashmead, 1900: 295; Szépligeti, 1904: 15; Vipio thoracica: Shenefelt, 1978: 1863.

#### Type material.

Holotype, ♀, *Glyptomorpha thoracica* Ashmead, 1900, **Grenada**: W.I. Chantilly Est. (Windward side) (no date) (H.H. Smith) (BMNH 3.c.540).

#### Additional material examined.

**Venezuela**: 1 ♀, Puerto Cabello, 10.i.1940 (P. Anduze) (USNM).

#### Diagnosis.

Raised area present on the face; T I with strong dorso-lateral carina; metasoma widely ovate and densely striate; hypopygium short; ovipositor length/body length 0.5; mesosoma dark reddish black.

#### Description.

**(females).** Length of body 4.8–6.2 mm, of fore wing 5.2–6.2 mm, of ovipositor (part exserted beyond apex of abdomen) 2.4–3.1 mm and of antenna 5.1–6.2 mm.

***Head.*** Antenna 1.0–1.1 × body length, with 39–45 flagellomeres; first flagellomere 2.0 × longer than wide, 1.5–1.6 × longer than 2^nd^, the latter 1.7 × longer than wide; median flagellomeres quadrate; terminal flagellomere acutely pointed apically; head transverse; face smooth; smooth and shiny; clypeus slightly rugulose, clypeal guard setae typical; HL 0.72–0.76 × HH; HW/HH 0.72–0.74; FH/FW 0.56–0.62; EH/HH 0.66–0.69 EH/FW 1.0–1.1; EW/EH 0.70–0.71; ITD 1.61–1.7 × TOD; MS 0.23–0.33 × EH; LMC 0.3 × HH; third segment of maxillary palpus 4.0 × longer than wide.

***Mesosoma.*** Length of mesosoma 1.74–1.8 × height; smooth and shiny; notauli smooth; propodeum smooth.

***Wings.*** Fore wing: length of fore wing 1.0–1.1 × body length; PL/LRC 0.84–0.97; PW/PL 0.22–0.27; length of vein 3RSb 0.86–0.97 × combined length of r-rs and 3RSa; length of vein 1M 0.68–0.71 × length of (RS+M)a; vein 3RSa reaching wing margin 0.69–0.71 × distance between apex of pterostigma and wing tip. Hind wing: uniformly setose; apex of C+SC+R with one basal hamule.

***Legs.*** Claw with pointed basal lobe.

***Metasoma.*** Metasoma widely ovate. First metasomal tergite 0.8–1.3 × longer than wide, raised median area oval, rugose; dorso-lateral carina laminate; T II 2.0–2.25 × wider than long, basal areas smooth, oblique furrows strongly impressed, crenulate; T III 2.2–2.6 × wider than medially long; T III–V longitudinally striate, T III and IV with anterolateral areas; T V–VII smooth and shiny; hypopygium ending at apex of abdomen; ovipositor 0.5 × body length.

***Colour.*** Face yellow, except a black, raised median area; palpi, tip of mandible, vertex, temple, occiput, and antenna black; metasoma reddish yellow to black. Wings smoky.

**Male.** Unknown.

#### Remarks.

This species resembles the Nearctic species *V. rugator* because of the presence of a raised area on the face, strong dorso-lateral carina of T I, widely ovate and densely striate metasoma, and short hypopygium. The relatively longer ovipositor (ovipositor length/body length 0.5) and reddish black mesosoma separate *thoracica* from *rugator*, in which the ovipositor is shorter (ovipositor length/body length 0.29–0.35) and the metasoma is yellow or reddish yellow.

**Figure 25.**
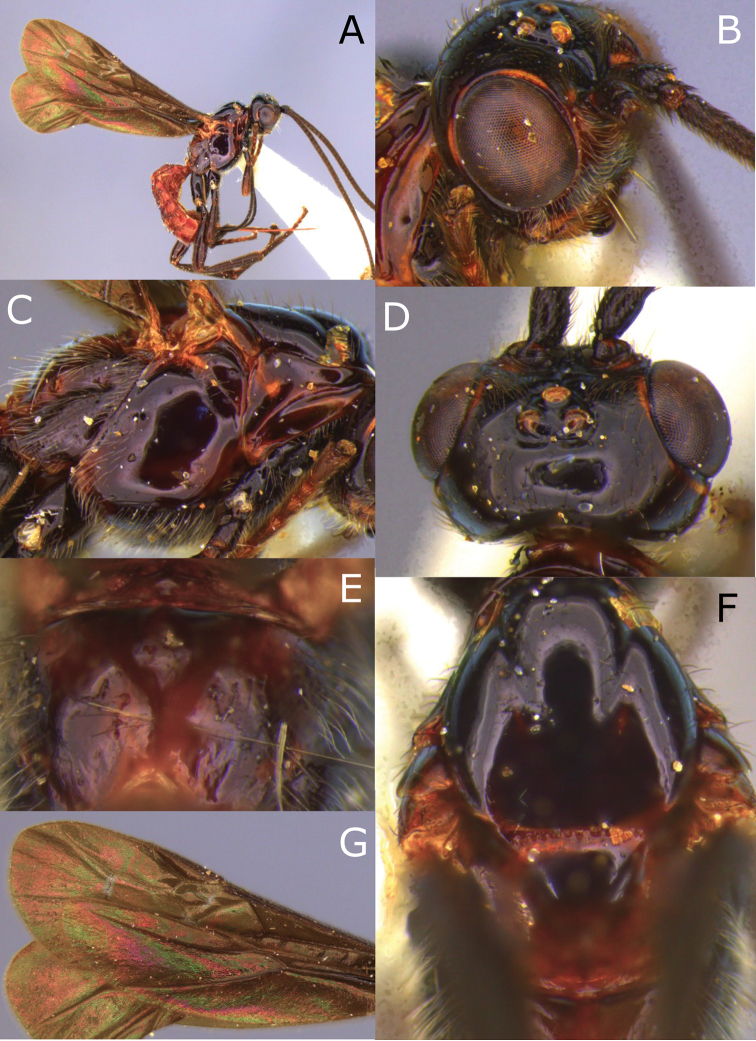
Montaged light micrographs of *Vipio thoracica*. **A** Holotype, habitus lateral view **B** head, oblique view **C** mesosoma, lateral view **D** head, dorsal view **E** propodeum **F** mesoscutum and scutellum, dorsal view **G** wings.

**Figure 26.**
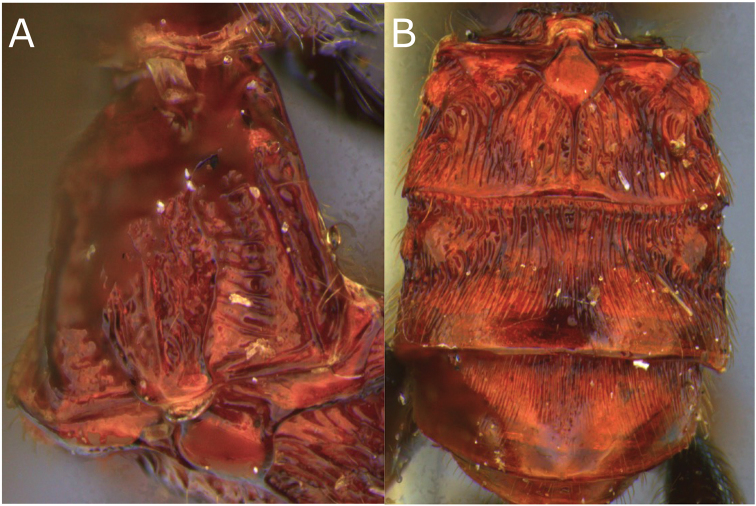
Montaged light micrographs of *Vipio thoracica*. **A** Holotype, metasomal tergite I, oblique dorsal view **B** holotype, metasomal tergites II–IV.

## Conclusions

The genus *Vipio* in the Neotropics is generally uncommon and most of the material available for examination is rather old. It can be noted that at the time of writing, not one Neotropical *Vipio* barcode sequence is listed on The Barcode of Life Data System (BOLD) (www.barcodinglife.org) (Ratnasingham and Hebert 2007) despite extensive Malaise trapping in Costa Rica, French Guiana, and Honduras, and examination of various other Neotropical samples. Currently, the BOLD database contains only 13 specimens of *Vipio*, one from a European Malaise trap and the remainder from an extremely extensive sampling of North America (Young et al. 2012, Steinke et al. 2017, Global Malaise Trap Program 2019). New and freshly collected material would be desirable for carrying out molecular investigation including DNA barcoding to test the current morphological taxonomic hypotheses.

## Supplementary Material

XML Treatment for Vipio

XML Treatment for Vipio belfragei

XML Treatment for Vipio boliviensis

XML Treatment for Vipio carinatus

XML Treatment for Vipio fiebrigi

XML Treatment for Vipio godoyi

XML Treatment for Vipio hansoni

XML Treatment for Vipio lavignei

XML Treatment for Vipio melanocephalus

XML Treatment for Vipio paraguayensis

XML Treatment for Vipio porteri

XML Treatment for Vipio quadrirugulosus

XML Treatment for Vipio strigator

XML Treatment for Vipio thoracica
